# Supplementary figures and images for: A simplified, combined protocol versus standard treatment for acute malnutrition in children 6–59 months (ComPAS trial): A cluster-randomized controlled non-inferiority trial in Kenya and South Sudan
Source: PLoS Med. 2020 Jul 9;17(7):e1003192. doi: 10.1371/journal.pmed.1003192 (PMC7347103; doi:10.1371/journal.pmed.1003192)

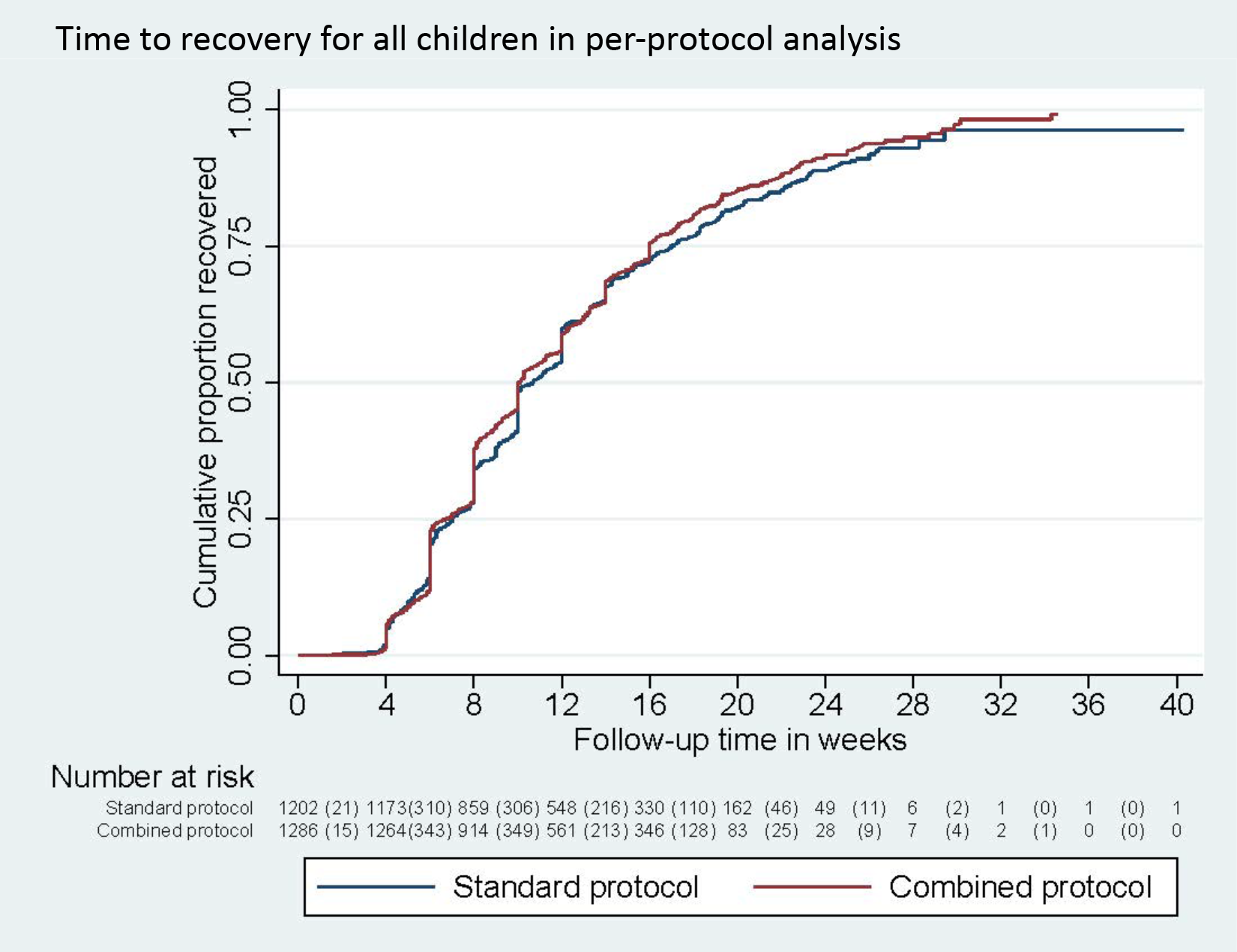

Supplement: S1 Fig — (TIF) [file pmed.1003192.s001.tif]

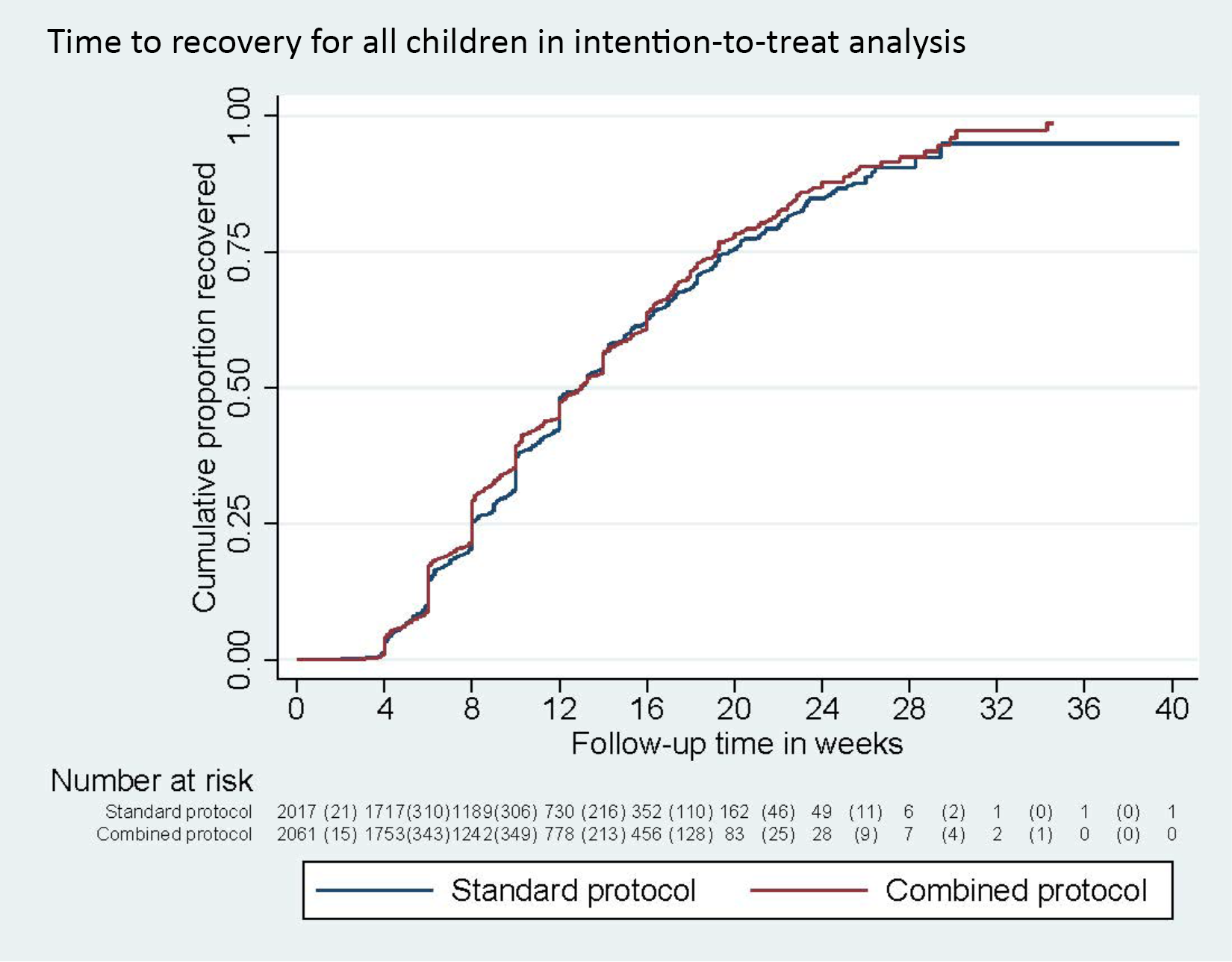

Supplement: S2 Fig — (TIF) [file pmed.1003192.s002.tif]

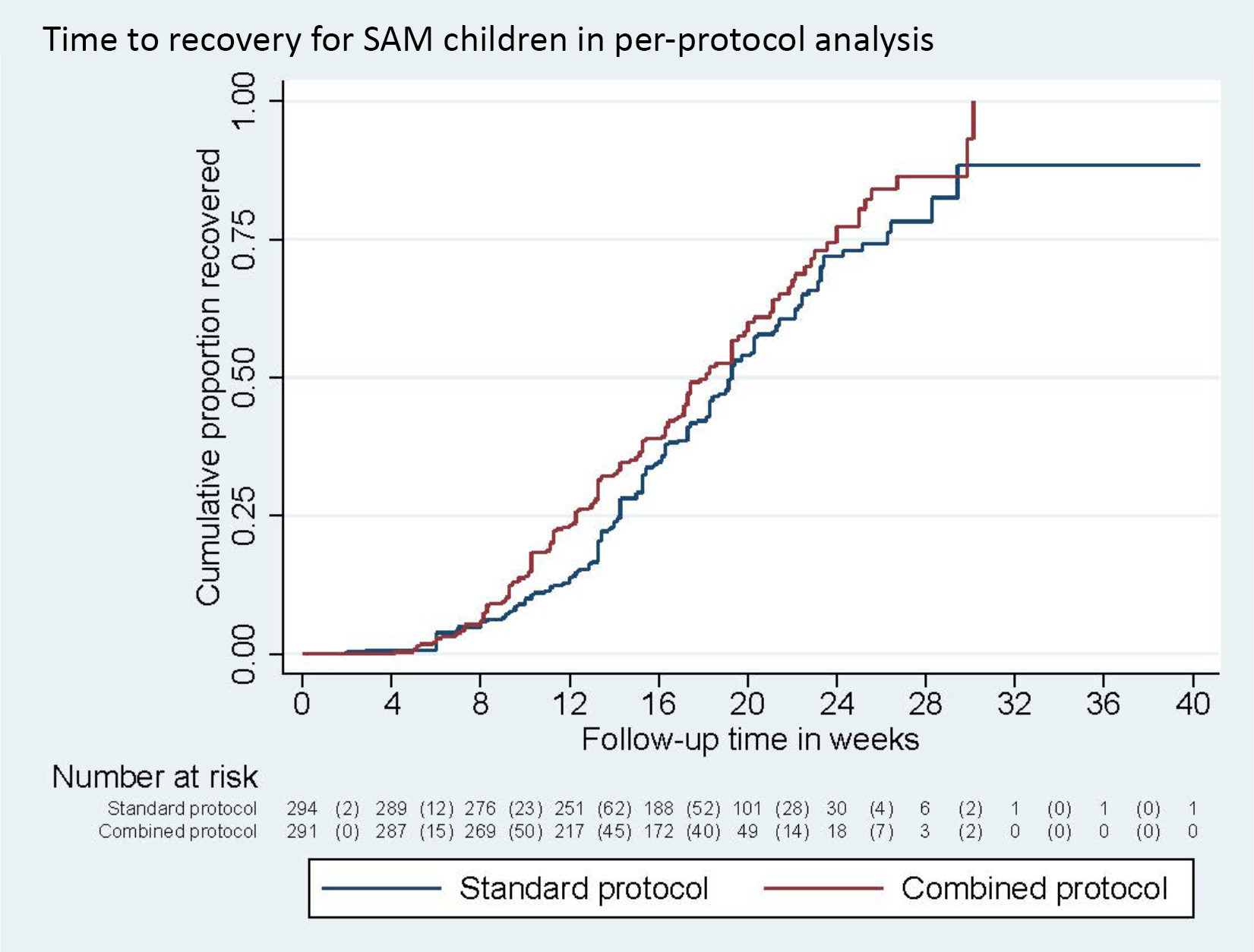

Supplement: S3 Fig — (TIF) [file pmed.1003192.s003.tif]

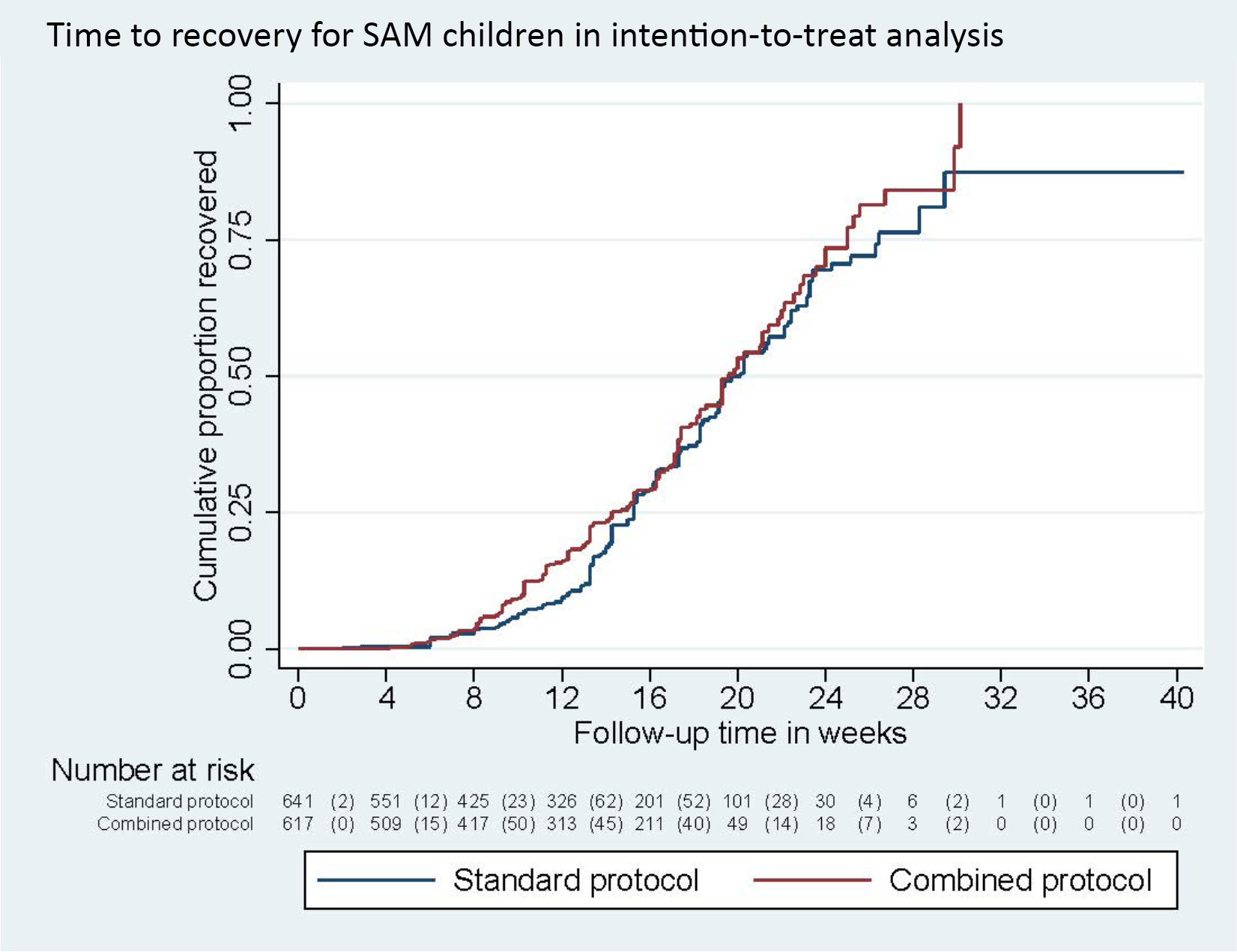

Supplement: S4 Fig — (TIF) [file pmed.1003192.s004.tif]

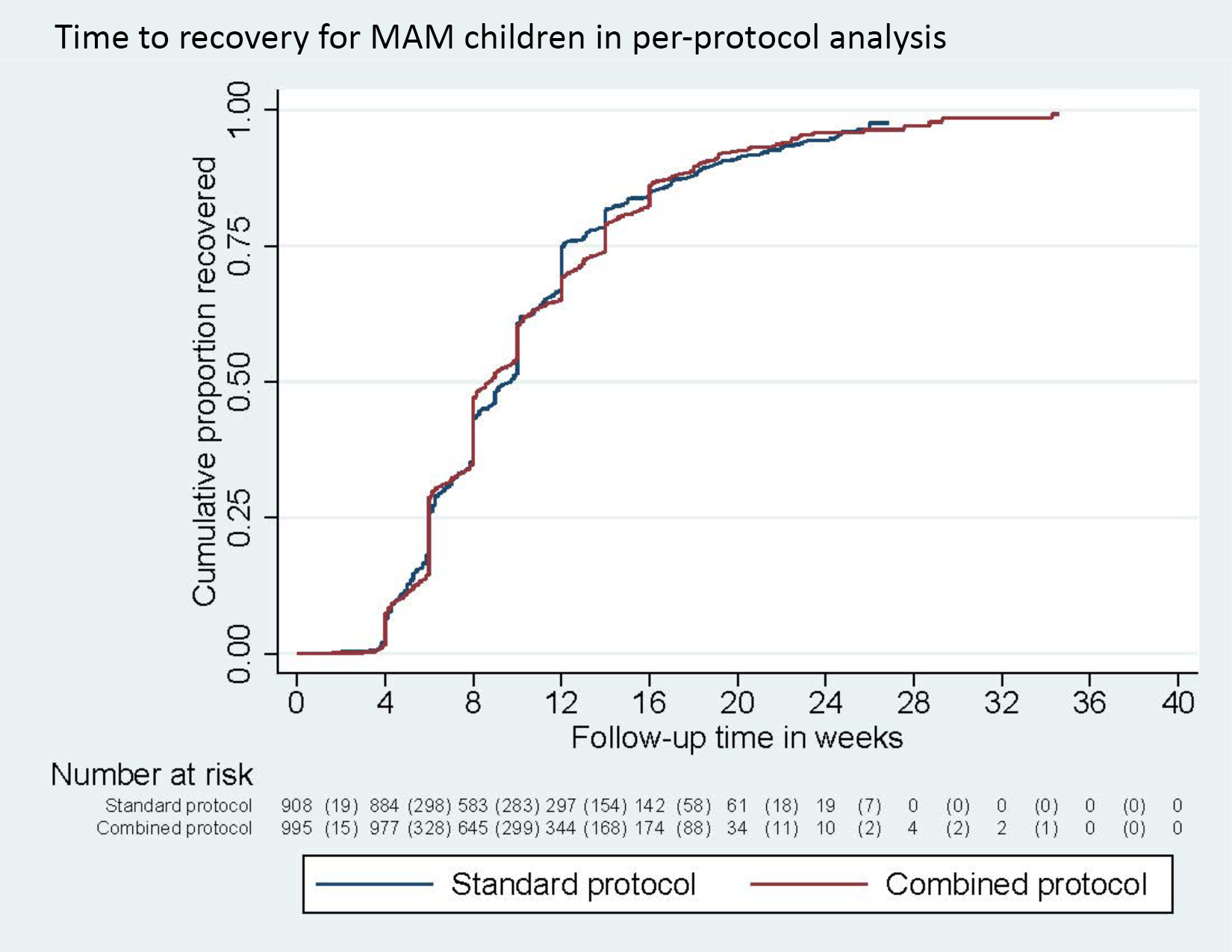

Supplement: S5 Fig — (TIF) [file pmed.1003192.s005.tif]

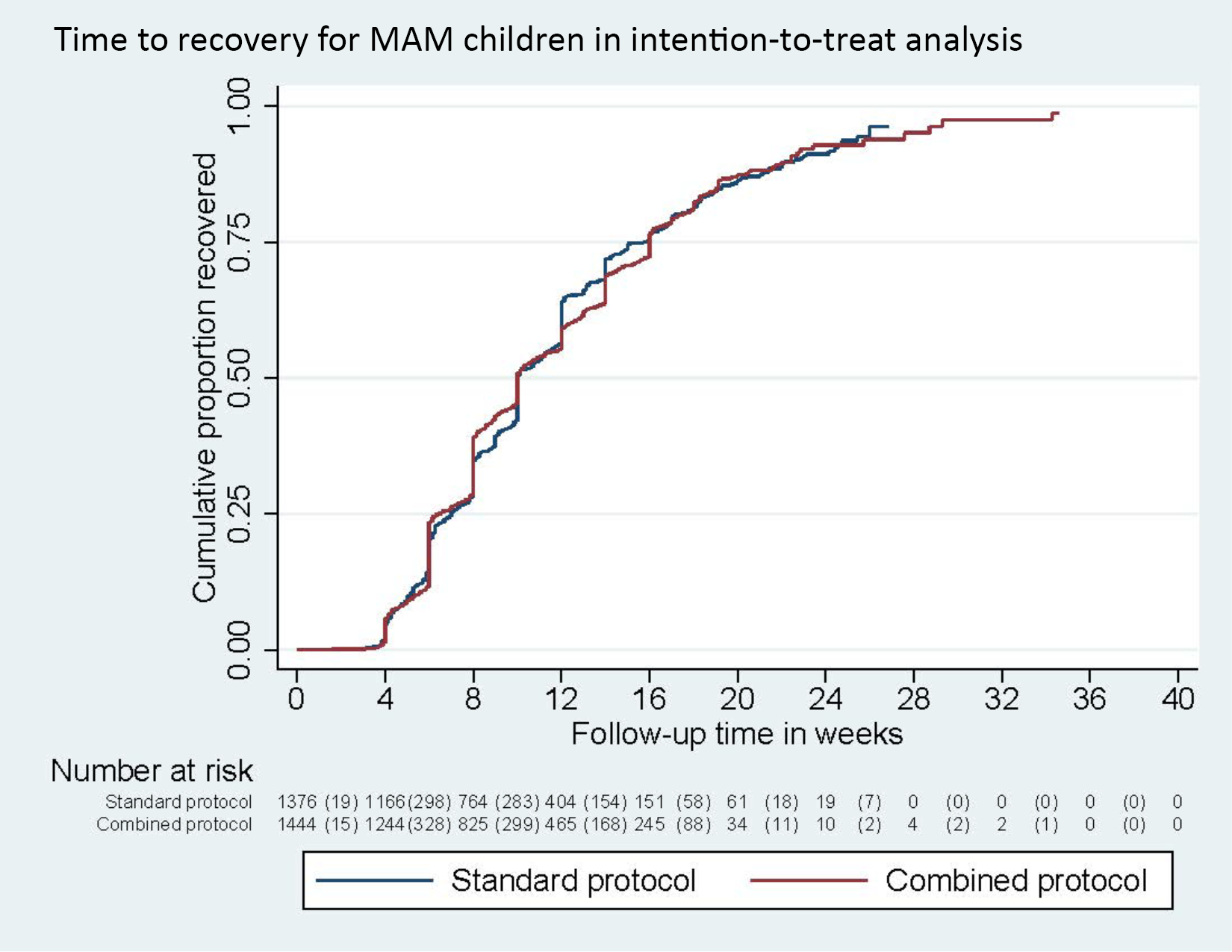

Supplement: S6 Fig — (TIF) [file pmed.1003192.s006.tif]
