# Supplementary material for: A simplified, combined protocol versus standard treatment for acute malnutrition in children 6–59 months (ComPAS trial): A cluster-randomized controlled non-inferiority trial in Kenya and South Sudan
Source: PLoS Med. 2020 Jul 9;17(7):e1003192. doi: 10.1371/journal.pmed.1003192 (PMC7347103; doi:10.1371/journal.pmed.1003192)
Supplement: S1 Table — (DOCX) [file pmed.1003192.s007.docx]

**Standard ready-to-use therapeutic food dosage table (based on 200 kcal/kg/day using 92g packets containing 500 kcal)**

| **Child’s weight (kg)** | **Packets per day** | **Packets per week** |
| --- | --- | --- |
| 4∙0*-4∙9 | 2 | 14 |
| 5∙0-6∙.9 | 2∙.5 | 18 |
| 7∙0-8∙4 | 3 | 21 |
| 8∙5-9∙4 | 3∙.5 | 25 |
| 9∙5-10∙.4 | 4 | 28 |
| 10∙5-11∙.9 | 4∙5 | 32 |
| ≥12 | 5 | 35 |

*Infants≥6 months and <4kg are referred to in-patient care
