# Supplementary material for: A simplified, combined protocol versus standard treatment for acute malnutrition in children 6–59 months (ComPAS trial): A cluster-randomized controlled non-inferiority trial in Kenya and South Sudan
Source: PLoS Med. 2020 Jul 9;17(7):e1003192. doi: 10.1371/journal.pmed.1003192 (PMC7347103; doi:10.1371/journal.pmed.1003192)
Supplement: S2 Table — (DOCX) [file pmed.1003192.s008.docx]

**Comparison of combined and standard protocols**

|  | **Standard Protocol**  **(CONTROL)** | **Combined Protocol**  **(INTERVENTION)** |
| --- | --- | --- |
| **Eligibility criteria** | Age 6-59 months, MUAC <12∙5cm and/or edema (+/++), and clinically uncomplicated (i.e. passes appetite test, no Integrated Management of Childhood Illness (IMCI) danger signs/ no serious medical complications) | Age 6-59 months, MUAC <12∙5cm and/or edema (+/++), and clinically uncomplicated (i.e. passes appetite test, no Integrated Management of Childhood Illness (IMCI) danger signs/ no serious medical complications) |
| **Admission criteria** | **OTP** | - <125mm MUAC   AND/OR   - Bilateral pitting edema (+/++)   AND   - clinically uncomplicated |
|  | - WHZ < -3   AND/OR   - MUAC < 115mm   AND/OR   - Bilateral pitting edema (+/++)   AND   - clinically uncomplicated |  |
|  | **SFP** |  |
|  | - Discharged from OTP   AND/OR   - WHZ <-2 to WHZ >-3   AND/OR   - MUAC 115mm- < 125mm   AND   - clinically uncomplicated |  |
| **Treatment frequency** | **OTP** | **MUAC <115mm and/or edema (+/++)** |
|  | Weekly | Weekly |
|  | **SFP** | **MUAC 115-<125mm** |
|  | 14 days | 14 days |
| **Treatment transition criteria** | - Child meets OTP ‘cured’ definition as described below | - Two consecutive MUAC measurements at or above 115mm   AND   - No edema |
| **Dosage** | **OTP** | **MUAC <115mm and/or edema (+/++)** |
|  | RUTF 200kcal/kg/day | RUTF 1000 kcal/day (2 sachets/day) |
|  | **SFP** | **MUAC 115-<125mm** |
|  | RUSF 500 kcal/day (1 sachet/day) | RUTF 500 kcal/day (1 sachet/day) |
| **Cured** | **OTP** | ≥125mm for 2 consecutive measurements and no edema |
|  | - Child maintains MUAC ≥115mm for two consecutive visits**   AND/OR   - WHZ >-3 Z-score for two consecutive visits**   AND   - No edema for two consecutive visits |  |
|  | **SFP** |  |
|  | Child maintains WHZ >-2 Z-score and/or MUAC ≥125mm for a period of two consecutive visits** |  |
