# Supplementary material for: A simplified, combined protocol versus standard treatment for acute malnutrition in children 6–59 months (ComPAS trial): A cluster-randomized controlled non-inferiority trial in Kenya and South Sudan
Source: PLoS Med. 2020 Jul 9;17(7):e1003192. doi: 10.1371/journal.pmed.1003192 (PMC7347103; doi:10.1371/journal.pmed.1003192)
Supplement: S3 Table — (DOCX) [file pmed.1003192.s009.docx]

**Admission characteristics of children by country**

| **Characteristic** | **Kenya (n=1,988)** | **South Sudan (n=2,122)** |
| --- | --- | --- |
| **Sex and age** | | |
| Males, n (%) | 742 (37%) | 940 (44%) |
| Age in months, mean (SD) | 12∙0 (7∙0) | 21∙7 (12∙0) |
| Age categories, n (%) |  |  |
| 6-<24 months | 1,880 (94∙6%) | 1,164 (54∙9%) |
| ≥ 24 months | 105 (5∙3%) | 954 (45∙0%) |
| **Anthropometrics** | | |
| Weight (kg), mean (SD) | 6∙8 (1) | 7∙7 (2) |
| Height (cm), mean (SD) | 69∙5 (6) | 76∙2 (10) |
| MUAC (cm), mean (SD) | 11∙8 (∙5) | 11∙6 (∙6) |
| WHZ, mean (SD) | -2∙1 (1∙0) | -2∙7 (3∙3) |
| HAZ, mean (SD) | -1∙7 (1∙5) | -2∙3 (1∙6) |
| WAZ, mean (SD) | -2∙5 (1∙0) | -3∙2 (1∙0) |
| MUAC <11∙5cm, n (%) | 354 (18%) | 881 (42%) |
| and WHZ ≥-3 to <-2 | 123 (35%) | 274 (31%) |
| and WHZ <-3 | 160 (45%) | 538 (61%) |
| and WHZ >=-2 | 71(20%) | 69(8%) |
| MUAC 11∙5-<12∙5cm, n (%) | 1,631(82%) | 1,227 (58%) |
| and WHZ ≥-3 to <-2 | 586 (36%) | 614 (50%) |
| and WHZ <-3 | 173 (11%) | 243 (20%) |
| and WHZ >=-2 | 872(54%) | 370(30%) |
| Edema (+ or ++), n (%) | 18 (1%) | 29 (1%) |
| **Household and other characteristics** | | |
| Mother is caretaker, n (%) | 1,915 (96%) | 1,986 (94%) |
| Maternal educational achievement, n(%) |  |  |
| None | 29(2%) | 1735(87%) |
| Pre-primary | 22(1%) | 0(0%) |
| Primary | 726(38%) | 234(12%) |
| Secondary | 1135(59%) | 16(1%) |
| College tertiary | 0(0%) | 1(0%) |
| Number of children under five in the home, mean (SD) | 1∙1 (∙7) | 2∙1 (∙8) |
| Children breastfed in last 24 hours, n(%) | 1,636 (82%) | 1,227 (58%) |
| Caretaker reports any morbidity in past week*, n (%) | 870 (44%) | 1,629 (77%) |
| Fever | 337 (17%) | 1,164 (55%) |
| Diarrhea | 275 (14%) | 763 (36%) |
| Cough | 479 (24%) | 866 (41%) |
| Healthcare sought in prior week | 367(19%) | 521(25%) |
| HIV Status |  |  |
| + | 14(1%) | 0(0%) |
| Exposed (Mother) | 36(2%) | 0(0%) |
| Disabled (physically or mentally) | 29(2%) | 53(3%) |
| Tuberculosis (+), n(%) | 9(1%) | 0(0%) |
| Access to toilet | 1978(100%) | 678(32%) |
| Water source |  |  |
| Household tap | 266 (13%) | 0(0%) |
| Community tap/ tap stand | 1381(70%) | 45(2%) |
| Hand pump/ borehole | 0(0%) | 1264(60%) |
| Borehole (private) | 26 (1%) | 588(28%) |
| Vendors | 304(15%) | 0(0%) |
| Open water | 1(0%) | 218(10%) |
| Livelihood/ main source of income |  |  |
| No income | 121(6%) | 1(0%) |
| Sale of items (grass, firewood, livestock) | 81(4%) | 340(16%) |
| Fishing/ farming | 0(0%) | 1208(57%) |
| Business/ shopkeeper | 821(41%) | 93(4%) |
| Casual labor | 425(21%) | 294(14%) |
| Salaried work | 31(2%) | 160(8%) |
| Other | 6(0%) | 19(1%) |
| Household Hunger Score |  |  |
| *Ever no food to eat?* |  |  |
| Never | 1541(78%) | 775(37%) |
| Rarely | 286(14%) | 653(31%) |
| Sometimes | 126(6%) | 590(28%) |
| Often | 23(1%) | 93(4%) |
| Don’t know | 2(0%) | 7(0%) |
| *Ever go to sleep without enough food?* |  |  |
| Never | 1560(79%) | 654(31%) |
| Rarely | 280(14%) | 753(36%) |
| Sometimes | 114(6%) | 631(30%) |
| Often | 21(1%) | 61(3%) |
| Don’t know | 3(0%) | 19(1%) |
| *Any HH member go whole day without eating anything?* |  |  |
| Never | 1581(80%) | 754(36%) |
| Rarely | 257(13%) | 688(32%) |
| Sometimes | 122(6%) | 578(27%) |
| Often | 16(1%) | 64(3%) |
| Don’t know | 2(0%) | 34(2%) |
