# Supplementary material for: A simplified, combined protocol versus standard treatment for acute malnutrition in children 6–59 months (ComPAS trial): A cluster-randomized controlled non-inferiority trial in Kenya and South Sudan
Source: PLoS Med. 2020 Jul 9;17(7):e1003192. doi: 10.1371/journal.pmed.1003192 (PMC7347103; doi:10.1371/journal.pmed.1003192)
Supplement: S4 Table — (DOCX) [file pmed.1003192.s010.docx]

**Coverage survey results by country, arm and SAM/MAM status**

|  | **Standard Protocol** | | **Combined Protocol** | |
| --- | --- | --- | --- | --- |
|  | **SAM** | **MAM** | **SAM** | **MAM** |
| **Kenya** (%)  (95% CI) | 52∙9%  (39∙1% - 66∙2%) | 47∙3%  (37∙1% - 58∙0%) | 54∙9%  (41∙2% - 68∙0%) | 48∙6%  (38∙0% - 59∙4%) |
| **South Sudan** (%)  (95% CI) | 62∙5%  (47∙8-75%) | 26∙3%  (19∙2-35∙1%) | 45∙9%  (32∙5-59∙9%) | 21∙3%  (14∙9-29∙2%) |

Definition of SAM: MUAC<11∙5cm and/or edema (+/++); definition of MAM: MUAC 11∙5-<12∙5cm and no edema
