# Supplementary material for: A simplified, combined protocol versus standard treatment for acute malnutrition in children 6–59 months (ComPAS trial): A cluster-randomized controlled non-inferiority trial in Kenya and South Sudan
Source: PLoS Med. 2020 Jul 9;17(7):e1003192. doi: 10.1371/journal.pmed.1003192 (PMC7347103; doi:10.1371/journal.pmed.1003192)
Supplement: S5 Table — (DOCX) [file pmed.1003192.s011.docx]

**Analyses of recovery and length of stay by sub-group**

| **Per Protocol Analysis** | **Standard Protocol**  **(N=12, n=1,202)** | | **Combined Protocol**  **(N=12, n=1,286)** | | **Unadjusted** | | **Adjusted** | |
| --- | --- | --- | --- | --- | --- | --- | --- | --- |
|  | **n** | **%** | **N** | **%** | **Risk difference^§^**  **(95% CI)** | **p-value** | **Risk difference^§^**  **(95% CI)** | **p-value** |
| **Recovery among sub-groups by admission status** | | | | | | | | |
| SAM | 111/294 | 37∙8% | 121/291 | 41∙6% | 0∙04 (-0∙06-0∙13) | 0∙43 | 0∙03 (-0∙05-0∙12) | 0∙42 |
| MAM | 773/ 908 | 85∙1% | 860/995 | 86∙4% | 0∙01 (-0∙07-0∙10) | 0∙77 | 0∙00 (-0∙07-0∙07) | 0∙97 |
| Age <24 months | 654/930 | 70∙3% | 667/928 | 71∙9% | 0∙02 (-0∙08- 0∙11) | 0∙75 | 0∙02 (-0∙07-0 ∙11) | 0∙62 |
| Age ≥24 months | 230/272 | 84∙6% | 314/358 | 87∙7% | 0∙03 (-0∙06-0∙13) | 0∙52 | 0∙04 (-0∙06-0∙13) | 0∙46 |
| SAM & ≥8kg | 29/54 | 53∙7% | 28/42 | 66∙7% | 0∙13 (-0∙08-0∙34) | 0∙23 | 0∙08 (-0∙14-0∙31) | 0∙47 |
| MUAC 11∙5-<12∙5cm and WHZ<-3z | 84/111 | 75∙7% | 134/190 | 70∙5% | -0∙05 (-0∙19-0∙08) | 0∙46 | -0∙05 (-0∙16-0∙07) | 0∙44 |
| Kenya | 516/686 | 75∙2% | 458/627 | 73∙1% | -0∙02 (-0∙13-0∙09) | 0∙71 | -0∙02 (-0∙13-0∙09) | 0∙70 |
| South Sudan | 368/516 | 71∙3% | 523/659 | 79∙4% | 0∙08 (-0∙03- 0∙19) | 0∙16 | 0∙08 (-0∙02-0∙18) | 0∙13 |
|  | **Standard Protocol**  **(N=12, n=1,201)** | | **Combined Protocol**  **(N=12, n=1,286)** | | **Unadjusted** | | **Adjusted** | |
|  | **Mean** | **SE** | **Mean** | **SE** | **Mean difference^§^**  **(95% CI)** | **p-value** | **Mean difference^§^**  **(95% CI)** | **p-value** |
| **Length of stay by country** | | | | | | | | |
| Kenya | 58∙9 | 2∙36 | 63∙5 | 2∙35 | 4∙62 (-2∙35-11∙59) | 0∙17 | 4∙73 (-2∙07-11∙52) | 0∙15 |
| South Sudan | 73∙5 | 2∙52 | 67∙0 | 2∙38 | -6∙45 (-13∙53-0∙63) | 0∙07 | -6∙37 (-13∙17- 0∙43) | 0∙06 |

**Unadjusted**: All children with outcome measures, not adjusted for any demographic or study design characteristics; **Adjusted**: for age, sex and country; **N**= number of clusters; **n**=number of children eligible for follow up; **^§^**standard errors adjusted for clustering within facilities
