# Supplementary material for: A simplified, combined protocol versus standard treatment for acute malnutrition in children 6–59 months (ComPAS trial): A cluster-randomized controlled non-inferiority trial in Kenya and South Sudan
Source: PLoS Med. 2020 Jul 9;17(7):e1003192. doi: 10.1371/journal.pmed.1003192 (PMC7347103; doi:10.1371/journal.pmed.1003192)
Supplement: S1 Text — (DOCX) [file pmed.1003192.s012.docx]

**Procedures for taking anthropometric measurements**

**Protocol for taking weight measurements:**

The child’s weight will be measured and documented at admission as well as at each follow-up visit.

1. Record weight using the ComPAS-designated SECA 385 scale, only
2. Ensure scale is on level floor or table top and set to zero (baby tray can be removed for children who are able to stand on their own)
3. Press ‘start’ button for first use or ‘reset’ button between each measurement; wait for ‘00∙0000’ to appear
4. Child removes any shoes and heavy clothing
5. Child is placed on tray or child stands on center of the scale, sitting/standing still (caregiver or clinician may have to persuade child to remain calm)
6. Clinician records measurement once reading is stable (digits will flash once and then stabilize)
7. Measurement is recorded to the nearest 0∙01kg

**Protocol for taking height/length measurements:**

The child’s height/length will be measured and documented at admission as well as on a monthly basis thereafter∙ Length is measured for those aged <2, height for those ≥2 years This measurement will be taken by two operators, and measurements compared.

1. Child should remove shoes and any hair ornaments that may interfere with reading
2. Child is laid or stands straight with heels against height/length board, toes directly in the air (feet at 90°angle standing position) or feet flat on floor
3. Ensure back, shoulders, head, buttocks are flat against height board and heels are flat against heel plate
4. Ensure the head in line with neck and shoulders (chin level)
5. Clinician moves foot board against child’s feet or head board onto child’s head
6. Clinician places hands under the subjects ears to assist with posture and then asks child to breath in and then relax but stay tall
7. Measurement is recorded to the nearest 0∙1cm
8. The two measurements should be within 0∙5cm of each other. If they are not, both operators should repeat the measurements. If they are within 0∙5cm of each other, the final value should be mid-way between the two (ie. added together and divided by 2)

**Protocol for taking MUAC measurements:**

The child’s mid upper arm circumference will be measured and documented at admission to determine eligibility for treatment as well as at each follow-up visit.

1. Shirt should be removed
2. Take the left arm of the child
3. Place the left arm of the child across his chest and ask the caregiver to keep the arm in this position.
4. Use the MUAC tool to determine the mid-point between the shoulder and tip (bone) of elbow
5. Strap the MUAC tool around the arm of the child at the identified mid-point, be sure tape is level and tension is correct (no skin bulging from the top or bottom of the MUAC tape)
6. With the arm of the child hanging down, record the digits that are shown at the arrow in the hole of the MUAC tool (round to the nearest 1 mm).
7. When recording on the form, use two digits to the left of the decimal place, and one digit to the right.

**Protocol for checking for oedema:**

A condition of bilateral pitting oedema (level + or ++) meets the criteria for admission into the study. Do not force the appearance of a pit by pushing too hard, especially with a fingernail. It is not accurate to report nutritional oedema if only one foot has oedema. Oedema level +++ should be immediately referred for inpatient care.

1. While child’s foot is limp, press firmly with the pad (not tip) of thumb on the top of the child’s feet for 5 seconds, then release
2. Check each for a pit or indentation that lasts as least 2 seconds after release
