# Supplementary material for: A simplified, combined protocol versus standard treatment for acute malnutrition in children 6–59 months (ComPAS trial): A cluster-randomized controlled non-inferiority trial in Kenya and South Sudan
Source: PLoS Med. 2020 Jul 9;17(7):e1003192. doi: 10.1371/journal.pmed.1003192 (PMC7347103; doi:10.1371/journal.pmed.1003192)
Supplement: S2 Text — (DOCX) [file pmed.1003192.s013.docx]

**Com**bined **P**rotocol for **A**cute **M**alnutrition **S**tudy (ComPAS)

Study Protocol

Version: 16 March 2017

**Good Clinical Practice (GCP)**

This trial will adhere to the principles outlined in the International Conference on Harmonisation Good Clinical Practice (ICH GCP) guidelines, protocol and all applicable local regulations.

**Audits and Inspections**

The study may be subject to audit by the London School of Hygiene & Tropical Medicine under their remit as sponsor, the Study Coordination Centre and other regulatory bodies to ensure adherence to GCP.

**List of Acronyms**

ACF Action Contre la Faim

cRCT Cluster-randomized Controlled Trial

CIFF Children’s Investment Fund Foundation

ComPAS Combined Protocol for Acute Malnutrition Study

CHW Community Health Worker

CMAM Community-based Management of Acute Malnutrition

CSB Corn-Soy Blend

DMC Data Monitoring Committee

ENN Emergency Nutrition Network

ECHO European Commission Humanitarian Aid Office

GCP..................................................................................................Good Clinical Practice

GNC Global Nutrition Cluster

IYCF Infant and Young Child Feeding

IMCI Integrated Management of Childhood Illness

ICMJE International Committee of Medical Journal Editors

IRC International Rescue Committee

LSHTM London School of Hygiene and Tropical Medicine

MSF Médecins Sans Frontièrs

MUAC Mid Upper Arm Circumference

MoH Ministry of Health

MAM Moderate Acute Malnutrition

MANGO Modelling an Alternative Nutrition Protocol Generalizable to Outpatient Care

NGO Non Governmental Organization

OFDA .USAID Office of U.S. Foreign Disaster Assistance

OTP Outpatient Therapeutic Program

PI Principal Investigator

RUSF Ready to Use Supplementary Food

RUTF Ready to Use Therapeutic Food

REC/IRB Research Ethics Committee/Institutional Review Board

SQUEAC Semi-quantitative Evaluation of Access and Coverage

SFP Supplementary Feeding Program

SAM Severe Acute Malnutrition

UNICEF The United Nations Children’s Emergency Fund

UN The United Nations

UNHCR United Nations High Commissioner for Refugees

USAID................................................United States Agency for International Development

WHZ Weight for Height Z-score

WFP World Food Programme

WHO World Health Organization

**Table of Contents**

[I. Administrative information 5](#_Toc453145666)

[1. Title 5](#_Toc453145667)

[2. Trial registration 5](#_Toc453145668)

[3. Protocol version 5](#_Toc453145669)

[4. Funding. 5](#_Toc453145670)

[5. Roles and responsibilities 5](#_Toc453145671)

[II. Introduction 8](#_Toc453145676)

[6. Background and rationale 8](#_Toc453145677)

[7. Objectives 11](#_Toc453145680)

[8. Trial design 12](#_Toc453145681)

[III. Methods: Participants, interventions, and outcomes 12](#_Toc453145682)

[9. Study setting 12](#_Toc453145683)

[10. Eligibility criteria 13](#_Toc453145684)

[11. Interventions 14](#_Toc453145685)

[12. Outcomes 18](#_Toc453145689)

[13. Participant timeline 19](#_Toc453145690)

[14. Sample size 20](#_Toc453145691)

[15. Recruitment 20](#_Toc453145698)

[IV. Methods: Assignment of interventions 21](#_Toc453145699)

[16. Allocation 21](#_Toc453145700)

[17. Blinding 21](#_Toc453145703)

[V. Methods: Data collection, management, and analysis 22](#_Toc453145706)

[18. Data collection methods 22](#_Toc453145707)

[19. Data management 24](#_Toc453145710)

[VI. Methods: Monitoring 24](#_Toc453145711)

[20. Data monitoring 24](#_Toc453145712)

[21. Harms 24](#_Toc453145715)

[VII. Ethics and dissemination 25](#_Toc453145716)

[22. Research ethics approval 25](#_Toc453145717)

[23. Protocol amendments 25](#_Toc453145718)

[24. Consent 25](#_Toc453145719)

[25. Confidentiality 25](#_Toc453145722)

[26. Declaration of interests 26](#_Toc453145723)

[27. Access to data 27](#_Toc453145724)

[28. Dissemination policy 27](#_Toc453145725)

[VIII. Appendices 28](#_Toc453145729)

A. Questionnaires for data collection

B. RUTF characteristics

[IX. References 29](#_Toc453145732)

# Administrative information

## Title Combined Protocol for Acute Malnutrition Study (ComPAS)

## Trial registration: ISRCTN 30393230

## Protocol version 16 March 2017

## Funding United States Agency for International Development/Office of Disaster Assistance (USAID/OFDA) and the Children’s Investment Fund Foundation (CIFF).

## Roles and responsibilities

### 5a Names, affiliations, and roles of protocol contributors (by group and in alphabetical order):

Global Principal and Co- Investigators

Jeanette Bailey, Principal Investigator, International Rescue Committee and London School of Hygiene and Tropical Medicine

Dr. André Briend, Professor, University of Tampere School of Medicine and University of Copenhagen

Dr. Marko Kerac, Clinical Lecturer, London School of Hygiene and Tropical Medicine.

Dr. Mark Manary, Professor, Washington University School of Medicine

Dr. Charles Opondo, Research Fellow, London School of Hygiene and Tropical Medicine.

South Sudan Co-Investigators

Dr. Samson Paul Baba, Director General for Primary Health Care, Ministry of Health, South Sudan

Pamela Onyoo, ComPAS Research Coordinator, Action Against Hunger-USA, South Sudan

Rebecca Alum William, Director of Nutrition, Ministry of Health, South Sudan

Kenya Co-Investigators

Bethany Marron, ComPAS Research Coordinator, International Rescue Committee, Kenya

Eunice Musyoki, ComPAS M&E Manager, International Rescue Committee, Kenya

IRC and ACF ComPAS Advisors

Dr. Lara Ho, Senior Advisor for Health Research, International Rescue Committee

Imelda Awino, Nutrition and Health Advisor, Action Against Hunger-USA

Global Consultative Committee

Erin Boyd, Nutrition Advisor, USAID Office of Foreign Disaster Assistance

Dr. Saskia de Pee, Senior Technical Advisor Nutrition, World Food Programme

Claire Harbron, Nutrition Advisor, Children’s Investment Fund Foundation

Diane Holland, Senior Nutrition Advisor, UNICEF

Lynnda Kiess, Team lead Nutrition-Specific Programming, World Food Programme

Grainne Moloney, Chief Nutrition, UNICEF Kenya

Dolores Rio, Senior Nutrition Advisor, UNICEF

Britta Schumacher, Team lead Nutrition in Emergencies, World Food Programme

Daniel Tewoldeberha, UNICEF Kenya

Sonia Walia, Health and Nutrition Advisor, USAID Office of Foreign Disaster Assistance

Marie Sophie Whitney, Nutrition Advisor, European Commission Humanitarian Aid Office

### 5b. Name and contact information for the trial sponsor

**Sponsor**

London School of Hygiene & Tropical Medicine will act as the main sponsor for this study.  Delegated responsibilities will be assigned locally.

Email contact: RGIO@lshtm.ac.uk

**Indemnity**

London School of Hygiene & Tropical Medicine holds Public Liability ("negligent harm") and Clinical Trial ("non-negligent harm") insurance policies which apply to this trial.

### 5c. Role of study sponsor and funders

The trial funders (OFDA, CIFF) were involved in choice of study sites and part of the consultative committee who were consulted about study design. They will however have no role in the collection, management, analysis, and interpretation of data; writing of the report; and the decision to submit the report for publication.

### 5d. Composition, roles, and responsibilities

The ComPAS study is managed by a **global research team** of members from the International Rescue Committee, ACF USA, and the MoH in South Sudan and Kenya. The IRC is the lead agency for the ComPAS study and the implementing partner with the MoH, UNICEF and WFP in Kenya, with ACF-USA as the implementing partner with the MoH, UNICEF and WFP in South Sudan. The LSHTM is the lead academic partner providing expertise in nutrition, statistics and epidemiology. ACF-UK is the prime holder of the CIFF grant and leader of the SAM 2.0 research consortium, of which ComPAS is a sub-grantee.

ComPAS is guided by an **expert scientific committee** including Dr. Marko Kerac (LSHTM), Dr. André Briend (University of Tampere and University of Copenhagen), Dr. Mark Manary (Washington University of St. Louis), and Dr. Charles Opondo (LSHTM), who helped interpret the data in Stage 1 and developed the treatment to be used in this study protocol. The expert scientific committee will make recommendations on any medical or epidemiological question that arises during the study design, implementation and interpretation of results.

ComPAS is also guided by a **global consultative committee** of senior nutritionists from The United Nations Children’s Emergency Fund (UNICEF), World Food Programme (WFP), USAID/OFDA, CIFF and European Commission Humanitarian Aid Office (ECHO). The consultative committee helped review and finalize this study protocol, and will play a key role in supporting the implementation of the study, as well as reviewing the results and proposing next steps at a policy level.

#

# Introduction

## Background and rationale

### 6a. Description of research question and justification for undertaking the trial

#### Description of research questions

IRC and ACF-USA, funded by OFDA and CIFF, supported by the LSHTM and in coordination the Ministries of Health in Kenya and South Sudan and will test a new nutritional protocol for the treatment of acute malnutrition that combines the treatment of Severe Acute Malnutrition (SAM) and Moderate Acute Malnutrition (MAM) to be implemented within the existing Community-based Management of Acute Malnutrition (CMAM) model. The goal of the Combined Protocol for the Treatment of Acute Malnutrition (‘Combined Protocol’) is to simplify and unify the treatment of severe and moderate acute malnutrition into one protocol in order to improve the global coverage, quality, continuity of care, and cost-effectiveness of acute malnutrition treatment.

Stage 1 of ComPAS began in October 2014. The specific objective of Stage 1 was to use observational data to analyze the growth of children recovering from acute malnutrition in OTP’s (Outpatient Therapeutic Program) and SFP’s (Supplementary Feeding Program) in order to assess energy requirements and propose an optimized dose of RUTF correlated with MUAC category. To do this, a statistical analyst examined data from anonymized databases provided by ACF-USA, Médecins Sans Frontières (MSF)-France and IRC, and observed the rate of growth and energy needs of children who ultimately achieved intended recovery goals. The results of the analysis were assessed by the ComPAS global team and the expert task force in London in January 2016 in order to develop this study protocol. The results from Stage 1 are presented at the end of section 6a (ii).

In Stage 2, the ComPAS study will address the following question:

What are the program quality indicators (recovery, defaulter, death, length of stay, average daily weight and Mid Upper Arm Circumference (MUAC) gain), and coverage following treatment under the Combined Protocol compared to the standard treatment of OTP and SFP?

#### Justification and summary of relevant studies

Prevalence surveys suggest that acute malnutrition affects approximately 7.5% of the world’s children under 5 years of age, with approximately 34 million children affected by MAM and 16 million children affected by SAM [^[[1]](#endnote-1)^]. Acute malnutrition is a continuum condition, but SAM and MAM are treated separately, with different protocols and therapeutic products managed by separate United Nations agencies. Although children with MAM are three times more likely to die than well-nourished children [^[[2]](#endnote-2)^], many nutrition programs offer treatment only of SAM due to resource and logistical constraints and the challenges of coordinating two separate programs.

There is global consensus around the use of Ready to Use Therapeutic Food (RUTF) for the treatment of SAM, but the treatment of MAM still lacks globally-accepted guidance. There have been many studies testing the efficacy or effectiveness of different products and supplements for the treatment of MAM, but the focus in MAM research has remained largely product-based with MAM regarded as a separate condition from SAM. The addition of milk protein to Ready to Use Supplementary Food (RUSF) appears to improve long-term outcome. A paper by Chang et al (2013) evaluating different products for the treatment of MAM demonstrated that sustained recovery was significantly more likely in those treated with soy + whey (RUSF) compared to either soy RUSF or corn-soy blend (CSB)++[^[[3]](#endnote-3)^]. During a 12-month follow-up period, only 63% of cured children remained well-nourished, 17% relapsed to MAM, 10% developed SAM, 4% died and 7% were lost to follow-up. Children treated with soy/whey RUSF were more likely to remain well-nourished (67%) than those treated with CSB++ (62%) or soy RUSF (59%) (P=0.01). RUTF for the treatment of children with SAM includes milk protein. Treating MAM with RUTF would provide MAM children with a high quality nutritional supplement, while reducing the logistics and cost of procuring a second product and managing a separate program for supplementary feeding.

The current protocol for SAM bases RUTF dosage on weight (175-200 kcal/kg/day) until children are discharged as cured, but children may not require as much energy towards the end of treatment. Ashworth (1969) demonstrates that in an inpatient setting, as children reach the discharge criteria, their food intake drops 30%, reflecting the concept that as children progress through treatment, their rate of growth slows and their supplemental energy needs decrease. One hypothesis suggests the composition of the tissue developed as children approach and then exceed discharge criteria changes from primarily lean muscle mass to fat mass, though more evidence is needed [^[[4]](#endnote-4)^]. Research from a MSF therapeutic feeding program in Burkina Faso indicates that average weight gain mirrors average MUAC gain and rate of growth using these two metrics slows as children progress through treatment, resulting in a growth ‘plateau’ where treatment gains stagnate [^[[5]](#endnote-5)^]. ACF-France in Myanmar successfully reduced dosage of RUTF to one sachet per day at the end of SAM treatment with similar programmatic results (cured/death/defaulter) as standard dosage of RUTF based on body weight [^[[6]](#endnote-6)^]. ACF is exploring a reduced dosage in SAM treatment through its ‘Modelling an Alternative Nutrition Protocol Generalizable to Outpatient’ (MANGO) research study in Burkina Faso in 2016-2017 [^[[7]](#endnote-7)^]_._

The use of MUAC as the sole anthropometric criteria for screening, admission and discharge has been shown to target those children most at risk for mortality. Rather than using WHZ as an additional criterion, greater sensitivity for the same specificity level for mortality can be achieved by increasing the MUAC cut-off [^[[8]](#endnote-8)^]. Data indicates that raising the MUAC cut-off to 125mm covers all children at risk of acute malnutrition-related deaths [^[[9]](#endnote-9)^]. MUAC-only programming is logistically easier to implement and more effective at detecting high risk children [^[[10]](#endnote-10)^] *.*

In 2013, Washington University initiated a clinical trial in Sierra Leone to test the efficacy of an integrated, MUAC-only based protocol for the treatment of SAM and MAM using one product (RUTF) at different doses for children <115mm (175 kcal/kg/day) and 115-<125mm (75 kcal/kg/day), against the standard protocol of OTP with RUTF for children admitted as SAM and SFP with CSB++ for children admitted as MAM. Children were discharged at a MUAC of 125mm with a package of preventive health care and Infant and Young Child Feeding (IYCF) support. Results from the trial indicate the integrated program had a reduced caseload of SAM, due to earlier treatment of children presenting as MAM, with a comparable recovery rate (83% vs. 79%) and higher coverage (71% vs. 55%, p=0.0005). Children who received integrated management recovered more rapidly, with greater MUAC gain and higher weight for height z- score (WHZ) upon discharge. The cost of RUTF used to treat a SAM case in the integrated program was $36, compared to $68 in the standard program [^[[11]](#endnote-11)^].

Building on the above mentioned studies, Stage 1 of ComPAS retrospectively analysed treatment data from 8,000 acutely malnourished children in five countries (Kenya, South Sudan, Chad, Yemen, and Pakistan) to assess growth trends and energy requirements.

Key findings from Stage 1 of ComPAS include the following:

- Growth trends in MUAC mirror those of proportional weight gain when examined by MUAC at last visit.
- Rates of weight and MUAC gain slow with increasing MUAC.
- Children with higher MUAC measurements need less energy per kilogram body weight to achieve the growth observed than those at lower MUAC measurements.
- When assessed over the range 115mm ≤ MUAC < 125mm, children treated in OTP facilities experienced more growth (defined by weight and MUAC gain) than children treated in SFP facilities
- When assessed by continent, children in Africa (Kenya, South Sudan and Chad) tended to gain weight and MUAC at a higher rate than children in Asia (Pakistan and Yemen). Rates of weight and MUAC gain slow at lower MUAC’s in children in Asia.
- When assessed by each stunting status (<-2 z-scores and ≥-2 z-scores) and age group (<2 years vs ≥ 2 years) at admission, no significant differences in weight and MUAC gain were detected between groups
- 1000 kilocalories a day meets the aim of covering the total energy needs of 95% of children when they have MUAC<115mm, and 500 kilocalories a day meets the aim of covering half the energy needs for 95% of children when they have 115mm ≤ MUAC < 125mm.

Our findings concluded that two 92g sachets of RUTF (1000 kcal) meets the total energy requirements for >95% of children with a MUAC<115mm, and one 92g sachet of RUTF (500 kcal) meets half the energy requirements for >95% of children with a MUAC of 115-<125mm, and serves to simplify and streamline the treatment provided in a combined protocol.

The Stage 1 report of ComPAS is included in annex.

### 6b. Explanation for choice of comparators

The Combined Protocol will be compared against the Standard Protocol (the national protocol in each country). The standard protocol includes treatment of SAM in an outpatient therapeutic program using RUTF (200 kcal/kg/day) and MAM in a supplementary feeding program using RUSF (500 kcal/day), as approved by WHO, UNICEF, WFP, and the Ministries of Health in each country. Both the Combined Protocol and the Standard Protocols for each country are described in detail in section 11.

## Objectives

Primary objective: Determine the impact of the Combined Protocol on recovery compared to the standard treatment of OTP and SFP (recovery defined as achieving ≥125mm MUAC).

Secondary objectives: Determine the impact of the Combined Protocol on coverage, defaulting, length of stay, death rate, average daily weight gain and average daily MUAC gain compared to the standard treatment of OTP and SFP.

Hypothesis: The treatment of acute malnutrition using the Combined Protocol will be as effective as the standard protocol at recovering children from malnutrition as indicated by program exit status, length of stay, and average weekly weight and MUAC gain. Coverage of SAM and MAM treatment will increase under a Combined Protocol by improving early detection of MAM and preventing deterioration into SAM, and by improving program coherence and reducing loss during the transition from OTP to SFP.

## Trial design

The study will be a prospective, multi-center cluster-randomized controlled (cRCT) non-inferiority trial. The unit of randomization will be health facilities stratified by country and then randomly assigned to the control or intervention group. Children in the control group will receive the standard protocol while those in the intervention group will receive the combined protocol.

The study will include a total of 24 clusters in the following categories:

Kenya

| Arm 1 | Control (6) | Standard protocol |
| --- | --- | --- |
| Arm 2 | Intervention (6) | Combined protocol |

South Sudan

| Arm 1 | Control (6) | Standard protocol |
| --- | --- | --- |
| Arm 2 | Intervention (6) | Combined protocol |

# Methods: Participants, interventions, and outcomes

## Study setting

**Description of study settings**

There will be two sites in this multi-center cluster randomized trial: Aweil East County, South Sudan and Nairobi county, Kenya.

Aweil East, South Sudan

Aweil East is a rural setting in Northern Bahr el Ghazal State which contains seven payams, a total population of 538,765 and an under five population of 102,365. It has a total of 13 government health facilities and outreach clinics in the area, all of which are supported by ACF-USA. Each health facility is approximately 20-30 km apart. 12 out of the 13 health facilities will be included in the study.

Nairobi, Kenya

Nairobi county is an urban area with 93 facilities offering treatment for malnutrition, a total population of approximately 3.4 million and an under five population of 450,000. Each treatment facility is approximately 3-5 km apart. 12 health facilities serving primarily internally displaced persons in 3 sub-counties of Nairobi will be selected.

## Eligibility criteria

Table 1: Study Inclusion and Exclusion Criteria

|  | **Inclusion** | **Exclusion** |
| --- | --- | --- |
|  | <125mm MUAC  *and/or*  Bilateral pitting oedema (+)  *and*  Passes appetite test (consumption of 30g of RUTF within 20 minutes)  *and*  No medical complications | ≥125mm MUAC  *or*  Failed appetite test (requires inpatient treatment)  *or*  Medical complications requiring inpatient treatment, as per international guidelines/ IMCI danger signs including but not limited to:   - Oedema (++ or higher) - Anorexia, no appetite - Intractable vomiting - Convulsions - Lethargy, not alert - Unconsciousness - Hypoglycaemia - High fever - Hypothermia - Severe dehydration - Lower respiratory tract infection - Severe anaemia - Skin lesions   *Note:*  For children with WHZ <-2 but a MUAC ≥125mm and no oedema:   - These children will be treated per the protocol provided at the health facility they present to. Their data will not be included in the primary analysis but reviewed in a secondary analysis. - For children presenting at a health facility allocated to the Combined Protocol:   - Children WHZ <-3 will receive two 92g sachets of RUTF/day. All medical aspects of treatment will be the same as the <115mm group.   - Children WHZ ≥-3 but WHZ <-2 will receive one 92g sachet of RUTF/day. All medical aspects of treatment will be the same as the 115mm-<125mm group. - For children presenting at a health facility allocated to the Standard Protocol:   - Treatment will be provided per the national protocol, which is inclusive of children with WHZ <-2.   Data will be collected on the total number of children screened, how many were included/excluded, and the reasons for exclusion from the study. |

Only health facilities that are registered MoH-run facility or mobile nutrition clinic sites will be included in the study. All staff will need to be official ACF, IRC or MoH staff trained in CMAM and the ComPAS study protocol.

## Interventions

### 11a. Interventions for each group

Table 2: Combined Protocol Intervention: Kenya and South Sudan

| **Combined Protocol** |  |
| --- | --- |
| **Admission Criteria** | <125mm MUAC  AND/OR bipedal oedema (+)  AND clinically uncomplicated (i.e. passes appetite test, no Integrated Management of Childhood Illness (IMCI) danger signs/ no serious medical complications) |
| **Treatment Frequency** | <115mm: Weekly  115-<125mm: Bi-weekly |
| **Dosage** | <115mm and/or oedema (+): Two 92g sachets RUTF/day (1000 kcal/day)  115-<125mm : One 92g sachet RUTF/day (500 kcal/day) |
| **Transition from 2 RUTF to 1 RUTF:** | Two consecutive weekly measurements at or above 115mm and no oedema |
| **Cured** | ≥125mm for 2 consecutive measurements and no oedema, with 3 week minimum stay |
| **Default** | Absent for 3 consecutive visits |
| **Non-recovered** | Has not achieved discharge criteria within 16 weeks |
| **Discharge procedures** | Discharge ration of 7 RUTF sachets |
| **Routine medical treatments (as per national protocol):** | For all children with MUAC <115mm and/or bipedal oedema (+) on admission:   - **Amoxicillin:** Give first dose at health facility and then give remainder to caretaker with instructions to give twice daily for 7 days   For all children with MUAC <125mm and/or bipedal oedema (+) on admission:   - **Malaria**: according to national protocol - **Measles:** one vaccine on 4^th^ visit (4^th^ week) (children >6 months) (unless already vaccinated) - **Deworming:** one dose (albendazole or mebendazole) on the second visit (second week) (children >1 year) |
| **Referral Procedures (as per national protocol)** | Any child who develops medical complications and/or is not responding to treatment will be referred for a medical evaluation and/or to the Stabilization Center.  ‘Not responding to treatment’ will be defined in the following way:   - Failure to gain any weight (non-oedematous children) - Failure to start to lose oedema - Oedema still present - Weight loss since admission to program (non-oedematous children) - Failure of appetite test - Weight loss of 5% of body weight - Weight loss for two successive visits |

Table 3- Standard Protocol Intervention- Kenya

| **Standard Protocol- Kenya** | **OTP** | **SFP** |
| --- | --- | --- |
| **Admission Criteria** | - WHZ < -3   And/or   - MUAC < 115mm   And/or   - Bilateral pitting oedema (+/++) | - Discharged from OTP   And/or   - WHZ <-2 to WHZ >-3  and/or - MUAC 115mm < 125mm |
| **Treatment Frequency** | Weekly | 14 days |
| **Dosage** | RUTF 200kcal/kg/day | - Plumpy Sup (500 kcal/day) |
| **Cured** | - MUAC >115mm   And/or  WHZ >-3 Z-score  and   - No oedema for two consecutive visits   and   - Minimum two months stay | Child maintains WHZ >-2 Z-score and/or MUAC ≥125mm for a period of two consecutive weighs/measurements  *For the purposes of analysis, children will be considered ‘cured’ after reaching ≥125mm for a period of two consecutive measurements.* |
| **Default** | Absent for 3 consecutive visits | Absent for 3 consecutive visits |
| **Non-recovered** | Has not achieved discharge criteria within 4 months | Has not achieved discharge criteria within 4 months |
| **Discharge procedures** | - Give a final ration of seven sachets as a weaning off ration - Refer to SFP | N/A |
| **Routine medical treatments** | - **Amoxicillin:** Give first dose at health facility and then give remainder to caretaker with instructions to give twice daily for 7 days - **Malaria**: according to national protocol - **Measles:** one vaccine on 4^th^ visit (4^th^ week) (children >6 months) (unless already vaccinated) - **Deworming:** one dose (albendazole or mebendazole) on the second visit (second week) (children >1 year) | N/A |
| **Referral Procedures** | Any child who develops medical complications and/or is not responding to treatment will be referred for a medical evaluation and/or to the Stabilization Center.  ‘Not responding to treatment’ will be defined in the following way:   - Failure to gain any weight (non-oedematous children) - Failure to start to lose oedema - Oedema still present - Weight loss since admission to program (non-oedematous children) - Failure of appetite test - Weight loss of 5% of body weight - Weight loss for two consecutive visits | |

Table 4- Standard Protocol Intervention- South Sudan

| **Standard Protocol- South Sudan** | **OTP** | **SFP** |
| --- | --- | --- |
| **Admission Criteria** | - WHZ < -3   And/or   - MUAC < 115mm   And/or   - Bilateral pitting oedema (+ /++)   AND   - Appetite test passed - No medical complications - Child clinically well and alert | - Discharged from OTP   And/or   - WHZ <-2 to WHZ >-3 and/or - MUAC 115mm < 125mm   AND   - Appetite test passed - No medical complications - Child clinically well and alert |
| **Treatment Frequency** | Weekly | 14 days |
| **Dosage** | RUTF 200kcal/kg/day | RUSF 500 kcal/day |
| **Cured** | Child maintains MUAC >115mm and/or WHZ >-3 and no oedema for two consecutive visits  Note: The anthropometric indicator (during admission) that is used to confirm SAM should also be used to assess whether a child has reached nutritional recovery (during discharge) | Child maintains WHZ >-2 and/or MUAC >125mm for two consecutive visits  and  6 week minimum stay  *For the purposes of analysis, children will be considered ‘cured’ after reaching ≥125mm for a period of two consecutive measurements.* |
| **Default** | Absent for 2 consecutive visits | Absent for 2 consecutive visits |
| **Non-recovered** | Has not achieved discharge criteria within 2 months | Has not achieved discharge criteria within 3 months |
| **Discharge procedures** | - Give a final ration of seven sachets as a weaning off ration - Refer to SFP | .N/A |
| **Routine medical treatments:** | - **Amoxicillin:** Give first dose at health facility and then give remainder to caretaker with instructions to give twice daily for 7 days - **Malaria**: according to national protocol - **Measles:** one vaccine on 4^th^ visit (4^th^ week) (children >6 months) (unless already vaccinated) - **Deworming:** one dose (albendazole or mebendazole) on the second visit (second week) (children >1 year) | - **Deworming:** one dose (albendazole or mebendazole) on the second visit (second week) (children >1 year) |
| **Referral Procedures** | Any child who develops medical complications and/or is not responding to treatment will be referred for a medical evaluation and/or to the Stabilization Center.  ‘Not responding to treatment’ will be defined in the following way:   - Failure to gain any weight (non-oedematous children) - Failure to start to lose oedema - Oedema still present - Weight loss since admission to program (non-oedematous children) - Failure of appetite test - Weight loss of 5% of body weight - Weight loss for two successive visits | |

### 11b. Criteria for discontinuing or modifying allocated interventions for a given trial participant

If staff or caretakers believe a child is not responding to treatment, they will be evaluated for medical complications. A more detailed clinical assessment with the potential for inpatient treatment will be available to all patients who develop medical complications and/or are not responding to treatment.

If a participant wishes to be removed from the study, their information will no longer be collected and they will be given the option to continue with the treatment they are currently receiving. If a participant does not wish to receive any treatment, counselling will be given to encourage the caretaker to keep the child in treatment until he/she is fully recovered.

**11c. Strategies to improve adherence to intervention protocols**

Participants will be given a one-on-one counselling session at admission to emphasize the importance of completing treatment, as well as practicing optimal hygiene and infant and young child feeding. Participants will be asked to return used RUTF packets on a weekly basis to monitor adherence and minimize the risk that RUTF packets are sold.

Trained community health workers will follow-up with absent patients to counsel caretakers who may be experiencing difficulties bringing their child to the clinic. Patients who miss 3 consecutive visits will be considered a defaulted participant. Children in both the intervention and control groups will be followed up in the same manner.

All participants in both the intervention and control groups will be referred to the nearest mother-to-mother or father-to-father support groups during and after treatment. The support groups offer health education and social support to caretakers of malnourished and at-risk children.

### 11d. Relevant concomitant care and interventions that are permitted or prohibited during the trial

Routine health care that can be treated on an outpatient basis will be provided to all children per the national CMAM protocol. Any child with a medical complication and/or not responding to treatment will be referred to the Stabilization Center for a medical evaluation. Inpatient care will be offered to those who require it for as long as needed. If a child is admitted for inpatient care, they will be discharged as a referral and exit from the study.

## Outcomes

Table 6: Primary and Secondary Outcomes

|  | **Measurement variable** | **Analysis metric** | **Method of aggregation** | **Time point** |
| --- | --- | --- | --- | --- |
| Primary | | | | |
| Recovery | MUAC ≥125mm and no oedema  Note: The definition of recovery in both the control and intervention groups will be defined as two consecutive measurements with a MUAC≥125mm and no oedema. For the purposes of analysis, children receiving the standard protocol will be considered as cured when their MUAC≥125mm and no oedema for two consecutive measurements, even if they continue to receive treatment until they reach a WHZ >-2. | Final value | Proportion | End of treatment |
| Secondary | | | | |
| Coverage | % of children eligible for treatment (MUAC<125mm) who receive it  Note: The coverage surveys will be based on a MUAC-only definition of Global Acute Malnutrition (GAM (MUAC<125mm). Weight and height measurements will not be taken. | Final value | Proportion | Semi-quantitative evaluation of access and coverage (SQUEAC) survey (after final enrolment) |
| Defaulter | Child discharged as defaulter (3 missed visits) | Final value | Proportion | End of treatment |
| Died | Child died during treatment | Final value | Proportion | End of treatment |
| Length of stay | Days in treatment | Duration of time | Sum | End of treatment |
| Average daily weight gain | grams/kilograms/day | Daily | Mean | End of treatment |
| Average daily MUAC gain | mm/day | Daily | Mean | End of treatment |

## Participant timeline

Once the study begins, all existing patients in the health facilities assigned to the intervention will complete treatment per the standard protocol, and all new patients eligible for inclusion will begin treatment per the Combined Protocol.

Enrolment: March- June 2017

SQUEAC: June 2017

Qualitative study: June 2017

End of study: October 2017

## Sample size

The primary outcome, ‘recovery’, in both the control and intervention groups will be defined as two consecutive measurements with a MUAC≥125mm and no oedema. We expect the recovery rate to be 85% based on the average program statistics in Nairobi, Kenya and Aweil East, South Sudan. If the combined protocol is non-inferior to the current protocol, allowing for a 10% non-inferiority margin, then we would require 12 clusters in each arm with 100 children in each cluster to demonstrate non-inferiority of the combined protocol, with 80% power at the 5% level of significance. An intracluster correlation coefficient (ICC) of 0.05 was used, a conservative estimate based on the results of a similar cluster randomized study testing an integrated SAM/MAM protocol in Sierra Leone (Maust et al. 2015).

In order to account for losses to follow-up (estimated as 15%) and cross-overs (estimated as 5% in each arm), 150 children per cluster will be recruited for inclusion in the study. This was determined based on the following calculation:

100 x 1/(1-.15) x (1/(1-.05-.05)²) = 146

Table 7: Sample Size Calculations

| **Assumptions** | **Value** |
| --- | --- |
| Power | .80 |
| Level of significance | .05 |
| Intervention recovery | .85 |
| Control recovery | .85 |
| Margin of Non-Inferiority | .10 |
| One or two-tailed test | 2 |
| Number of facilities per arm | 12 |
| **Individuals per cluster** | **100** |
| **Total individuals** | **2400 (1200 in South Sudan, 1200 in Kenya)** |

## Recruitment

Prior to randomization of health facilities to either the control or intervention group, community health workers will conduct a sensitization campaign across the communities that fall within the catchment area of the 12 facilities in each country. The goal of the campaign will be to meet with community leaders and encourage them to send malnourished children to the clinics for treatment. Randomization of the facilities will occur prior to the start of the trainings of the field teams, as research officers will only be trained on the protocol they are to implement. Screening to detect malnourished cases will take place after the randomization, since referral of children to the facilities cannot begin until the teams are trained and in place and ready to provide services. Community health workers will conduct screenings in an equivalent manner across the catchment areas of both arms of the study. All malnourished children (i.e. MUAC <125mm) will receive a referral slip and their caretaker will receive instructions on how to access treatment. For any medically complicated cases requiring immediate treatment, IRC/ACF will provide transportation to the nearest stabilization center.

Patients who present to health facilities on their own will also be screened for inclusion. Weight and height measurements will be taken at the health facility only (in addition ot MUAC measurements), per national and international guidelines. All patients eligible to participate in the study based on the inclusion and exclusion criteria will be invited to enrol in the study.

In Nairobi, Kenya in 2015 the average monthly enrolment at each health facility of children classified with acute malnutrition according to the primary study definition of <125mm MUAC is 30. It is expected it will take four months to reach the sample size in Nairobi.

In Aweil East, South Sudan in 2015 the average monthly enrolment at each health facility of children classified with acute malnutrition according to the study definition of <125mm MUAC is 80. It is expected it will take two months to reach the sample size in Aweil East.

Using the above averages, enrolment is expected to take a minimum of four months, with an additional 4 months for completion of treatment of the last enrolled child. Therefore the study is expected to take 8 months.

Enrolment will occur over a period when malnutrition tends to be at its highest.

# Methods: Assignment of interventions (for controlled trials)

## Allocation

### 16a. Sequence generation

Clusters (health facilities) will be stratified according to country (Kenya and South Sudan) and then randomly assigned to the intervention or control group according to a computer-generated allocation sequence.

### 16b. Implementation

The research coordinator in each country will conduct the randomization exercise, and will inform the research teams after the interventions are assigned. Participants will be enrolled by the research officer located at each health facility. Health facilities will be randomized, not individuals.

## Blinding (masking)

There will be no blinding in this trial as it is not logistically possible.

# Methods: Data collection, management, and analysis

## Data collection methods

### 18a Plans for assessment and collection of outcome, baseline, and other trial data

A dedicated research officer will be based at each health facility for the entire duration of the study. All research officers will be given a two week training prior to study inception. They will be required to pass an exam after training is complete to test protocol knowledge, anthropometric measurement techniques (weight, height, and MUAC measurements and oedema assessment), data management techniques and coaching and counselling methodologies.

Each research officer will oversee the training and supervision of health facility staff, enrolment and treatment of children according to the study protocol and data management. S/he will conduct initial and refresher trainings with health facility staff as well as have continuous on the job trainings. They will assess the accuracy of anthropometric measurements taken by health facility staff and review the patient cards and registers on a daily basis to ensure data quality.

The information below will be collected in addition to the patient cards used in each country. MoH OTP cards will be used for all participants in the Combined Protocol. MoH OTP or SFP cards (as applicable) will be used for all participants in the Standard Protocol. Forms will be pilot tested and where needed adjusted prior to the start of the main study.

1. **Admission** (in addition to the patient card)

- Mother is caretaker (yes/no)
- Father lives in home (yes/no)
- Maternal educational achievement (none, primary, secondary)
- # children <5 in household
- Access to toilet
- Water source
- Breastfeeding in last 24 hours (yes/no)
- Livelihood/main source of income
- Other sources of free food (blanket feeding, general food distribution, etc)
- Household Hunger Score:
  - Was there ever no food to eat of any kind in your house?
  - Did you or any household member go to sleep at night and there was not enough food?
  - Did you or any household member go a whole day without eating anything at all because there was not enough food?

1. **Weekly** (in addition to the patient card) :
   - Presence of morbidity since last visit (diarrhea, vomiting, fever, cough)- *SFP*
   - Referral for medical evaluation since last visit
   - Breastfeeding in last 24 hours (yes/no)
2. **Discharge** (in addition to the patient card)
   - Breastfeeding in last 24 hours (yes/no)
   - Questions to assess opportunity cost of accessing treatment to contribute to the cost-effectiveness analysis
3. **SQUEAC** (after completion of final enrolment)

A Semi-quantitative evaluation of access and coverage (SQUEAC) will be conducted at the mid-point of the trial to assess coverage in each health facility catchment area. The SQUEAC methodology has been validated by the Coverage Monitoring Network (CMN) and is the preferred coverage assessment methodology for comparison across nutrition programs. ComPAS will use a consultant from the CMN to lead the SQUEAC coverage surveys in both Kenya and South Sudan. The results will be per health facility catchment area. The coverage surveys will be based on a MUAC-only definition of Global Acute Malnutrition (GAM (MUAC<125mm). Weight and height measurements will not be taken.

The numerator/denominator for the SQUEAC coverage survey will be:

# of children who receive treatment for acute malnutrition/# of children eligible for treatment (<125mm MUAC)

1. **Qualitative end-line study** (focus group discussions and key informant interviews)

- Perceptions of program (caretaker acceptability and preferences)
- Sharing and sale of RUTF

The data collection matrices for Kenya and South Sudan can be found in annex.

### 18b. Plans to promote participant retention and complete follow-up

Participants will be given a careful explanation of the importance of completing treatment and have the opportunity to ask any questions prior to enrolment. As described in section 21, all participants will be given the contact information of two local representatives they can approach if they have any feedback for or wish to express any concerns at any time during the study. A health visitor (a community health worker) will follow-up patients who begin to default from treatment (after one week of missed treatment) to assess the reasons for defaulting and help the family strategize on ways to complete to treatment.

If a family chooses not to bring their child back to complete treatment, the child will be discharged as ‘defaulted’ after 3 weeks of missed treatment. No additional data will be collected on children who default.

## Data management

The IRC in Kenya and ACF in South Sudan will use CommCare <https://www.commcarehq.org/home/> to digitally collect all patient info on android tablets. All data will be captured both on paper and digitally, in order to allow for cross-checking of information.

Data on children in the combined protocol will be collected using the OTP patient card, and additional study data will be collected on separate forms for admission, weekly follow-up visits and discharge. Data on children in the standard protocol will be collected using either the OTP or SFP patient card (as appropriate according to anthropometric classification), and additional study data will be collection on separate forms for admission, weekly follow-up visits and discharge. A research officer will be based full-time at each of all 24 health facilities in the study, and will be responsible for entering the information from the patient cards and the additional forms into the CommCare system for each study participant.

Each week, the research coordinator will review all data, and will cross-check a sample of paper forms against the data in CommCare. All discrepancies will be verified against the paper records and the database corrected, as needed. The principal investigator will review the data uploaded into the database on a weekly basis and will follow-up with the research coordinators in Kenya and South Sudan with any questions or concerns.

All patient files will be maintained securely in a locked file throughout the program to ensure patient confidentiality. Unique identifiers will be assigned to each patient and name/address, etc will not be included in the research database used for analysis. The database will be maintained by the PI in a password-protected server hosted by the International Rescue Committee. Only co-investigators (those listed as part of the research team) will have access to the database, with access managed by the PI, until final analyses are complete. After analysis is complete, the final (anonymized) database will be made available on a data repository for further research purposes.

# Methods: Monitoring

## Data monitoring

### 20a Role of Data Safety and Monitoring Board (DSMB)

It is expected the trial enrolment will be complete within 3-4 months, due to high caseloads in the catchment area of each health facility. Due to the short duration of the trial (8 months, including 4 months for completion of treatment of the last enrolled child), meaningful interim analysis by a DSMB will be difficult. However, in order to have independent oversight of the safety aspects of the trial, a Trial Safety Committee (TSC) will be formed. The TSC will comprise of an independent chair, a statistician from the LSHTM, and a health official from each country (Kenya and South Sudan).

### 20b Description of any interim analyses and stopping guidelines, including who will have access to these interim results and make the final decision to terminate the trial

The TSC will review the study outcomes and adverse events at the mid-point of the trial to determine if the trial can proceed. Adverse events will include deaths and hospitalizations.

## Harms

**Plans for collecting, assessing, reporting, and managing solicited and spontaneously reported adverse events and other unintended effects of trial interventions or trial conduct**

The RUTF used in this study will be stored according to USAID guidelines outlined in appendix B. When stored at 26.7°C (80°F), RUTF has a shelf life of 24 months. The expiry date of RUTF used in this trial will be monitored by the ACF and IRC drug management teams in each location. RUTF in South Sudan will be stored in ACF warehouses currently used to store RUTF for existing programs in Aweil East, and RUTF in Kenya will be stored in IRC warehouses in Nairobi currently used to store RUTF prior to shipment to field programs.

The trial safety committee will review all cases of children who were hospitalized or who died at the trial mid-point to determine if these cases were study-related. Any cases deemed to be study-related will be reported to the PI and co-investigators, who will discuss with the IRB at LSHTM and in-country.

During the enrolment process each participant’s caretaker will be provided with the contact information for a point person who will be responsible for fielding complaints and concerns. The point person will not be the research coordinator or the research officer based at the health facility, but a third party representative who is not a part of the research team and can field concerns neutrally and without the participant fearing a loss of access to treatment. The point person will bring concerns to the research coordinator maintaining the anonymity of the study participant(s). The research coordinator will raise the issues with the PI and study team in order to find a resolution.

Additionally, the Community Health Workers (CHWs) employed by the project will organize monthly focus group discussions at the community level to understand perceptions of the program and solicit advice and feedback.

# Ethics and dissemination

## Research ethics approval

This study protocol has been approved by the following ethical review committees:

1. Kenya Medical Research Institute (KEMRI), Nairobi, Kenya
2. Ministry of Health Ethical Committee, Juba, South Sudan
3. LSHTM, London, United Kingdom

## Protocol amendments

Any important protocol modification (including but not limited to any changes to the treatment or those eligible for it, and/or how data will be collected, managed and reported) will be described in an amendment to this document and re-submitted to the three REC/IRB’s listed in section 24.

## Consent or assent

As this is a cluster randomized trial, consent will be obtained at the health facility level from local health authorities as well as from individual participants.

Health facility-level consent will be discussed between the research coordinator, community leaders and the MoH in each county. Community leaders and local health authorities designated by the MoH will receive a ‘health facility information sheet,’ and will be asked for their consent after they have had an opportunity to review the information and discuss any questions with the research coordinator.

When a caretaker arrives to any health facility included in the ComPAS trial seeking treatment for their malnourished child (either with a referral slip or not), they will first be seen by a community health worker to confirm that they are eligible to receive treatment (i.e., they have a MUAC <125mm, and/or a WHZ <-2 z-scores, and/or oedema). Once their eligibility for treatment is confirmed, they will be seen by the health worker responsible for their care. The health worker will describe the aims of the study as detailed in the ‘participant information sheet,’ ensuring that the caretaker feels comfortable and understands the information. They will be informed that a study is taking place at this health facility and will be given information about what their participation would entail, including how their information will be used, but that they are not obligated to participate. The participant information sheet provides details of the treatments provided in the study. Treatment will be available for all malnourished children who meet the national standards (per the eligibility criteria described above and in the interventions section of the study protocol) regardless of whether they choose to participate in the study or not. If a caretaker has chosen not to participate in the study, they will be assured that their child can still receive the treatment offered by that health facility, and that their information will not be included in the analysis.

## Confidentiality

Personal information (child’s name, address and age and a unique ID) will be included in the database housed at field level until treatment is complete and all data cross-checked. Name, address and age will be removed from the database before analysis, with only the unique ID remaining. Only co-investigators (those listed as part of the research team) will have access to the database.

No personal information will be shared for research purposes. The list of personal information will be used solely to track children for counselling to ensure completion of treatment, and to ensure data quality. After the close of the study and before final analysis all personal information will be deleted.

## Declaration of interests

No conflict of interest is declared by the principal investigator.

## Access to data

The final trial dataset will be made available to the institutions involved in implementing, funding or supporting the research:

- MOH, Kenya
- KEMRI, Kenya
- MOH, South Sudan
- Drug and Food Control Authority, South Sudan
- National Bureau of Statistics, South Sudan
- ComPAS expert committee
- IRC
- ACF-USA
- ACF-UK
- LSHTM
- USAID/OFDA
- CIFF
- WFP
- UNICEF
- WHO

## Dissemination policy

### 28a Plans for investigators and sponsor to communicate trial results to participants, healthcare professionals, the public, and other relevant groups

The final trial results will be written into a final report and shared with the institutions described in section 27. The interpretation of results, implications and next steps will be discussed with the MoH in each country, the expert committee and the consultative committee before dissemination or publication at either national or international levels.

A presentation will be made at the Nutrition Information Technical Working Group and the Emergency Nutrition Advisory Committee in Nairobi, Kenya and at the MoH and Nutrition Cluster in Juba, South Sudan to review the finalized results with key national stakeholders and discuss next steps for dissemination and publication.

Results will also be shared with key global nutrition committees and working groups, including the Global Nutrition Cluster (GNC), the Emergency Nutrition Network (ENN) and the Nutrition Forum, among others.

An article for publication in a peer-reviewed journal will be prepared summarizing the key outcomes of the trial and the implications for nutrition program policy. A separate article will be prepared summarizing the key outcomes of the cost-effectiveness analysis.

A workshop will be organized within one year of the trial end date to present the finalized analyses and discuss the policy implications. The workshop will be open to all key stakeholders (Non Governmental Organizations(NGO), donors, the United Nations (UN), Ministries of Health, universities) involved in the ComPAS study.

### 28b Authorship eligibility guidelines

International Committee of Medical Journal Editors (ICMJE) guidelines for authorship eligibility will be followed. No professional writers will be used.

Per the ICMJE, authorship will be based on the following four criteria:

- Substantial contributions to the conception or design of the work; or the acquisition, analysis, or interpretation of data for the work; AND
- Drafting the work or revising it critically for important intellectual content; AND
- Final approval of the version to be published; AND
- Agreement to be accountable for all aspects of the work in ensuring that questions related to the accuracy or integrity of any part of the work are appropriately investigated and resolved.

### 28c Plans for granting public access to the full protocol, participant-level dataset, and statistical code

The study protocol and statistical code will be made available to the public. The participant-level database will only be shared with the institutions listed in section 29.

# Appendices

Appendix A: Questionnaires for data collection

Appendix B : RUTF characteristics

# References

1. Levels and Trends in Child Malnutrition: UNICEF-WHO-The World Bank Joint Child Malnutrition Estimates. Washington D.C; 2015 [↑](#endnote-ref-1)
2. Matilsky D, Maleta K, Castleman T, Manary, M. Supplementary Feeding with Fortified Spreads Results in Higher Recovery Rates Than with a Corn/Soy Blend in Moderately Wasted Children. Journal of Nutrition. February 2009. [↑](#endnote-ref-2)
3. Chang C, Trehan I, Wang R, Thakwalakwa C, Maleta K, Deitchler M, Manary M. Children successfully treated for moderate acute malnutrition remain at risk for malnutrition and death in the subsequent year after recovery. Journal of Nutrition. 2013. [↑](#endnote-ref-3)
4. Ashworth, A. Growth rates in children recovering from protein-calorie malnutrition. British Journal of Nutrition. (1969), 23,835 [↑](#endnote-ref-4)
5. Goossens S, Bekele Y, Yun O, Harczi G, Ouannes M, Shepherd S (2012). Mid-Upper Arm Circumference Based Nutrition Programming: Evidence for a New Approach in Regions with a High Burden of Acute Malnutrition. PLoS ONE 7(11). [↑](#endnote-ref-5)
6. Qualitative review of an alternative treatment of SAM in Myanmar. Field Exchange. Issue 42 (January 2012) <http://fex.ennonline.net/42/qualitative>. [↑](#endnote-ref-6)
7. <http://www.isrctn.com/ISRCTN50039021> [↑](#endnote-ref-7)
8. Briend, A et al. Low mid-upper arm circumference identifies children with a high risk of death who should be the priority target for treatment. Response to Editor. BioMed Central Nutrition. 2016 (Not yet published). [↑](#endnote-ref-8)
9. Myatt M, Khara T, Collins S. A review of methods to detect cases of severely malnourished children in the community for their admission into community-based therapeutic care programs. Food Nutr Bull. 2006;27(3 Suppl):S7-23 [↑](#endnote-ref-9)
10. Briend A, Maire B, Fontaine O, Garenne M. Mid-upper arm circumference and weight-for-height to identify high-risk malnourished under-five children. Matern Child Nutr. 2012;8:130–3. [↑](#endnote-ref-10)
11. Maust A, Koroma A, Abla C, Molokwu N, Ryan K, Singh L, Manary M. Severe and Moderate Acute Malnutrition Can Be Successful Managed with an Integrated Protocol in Sierra Leone. Journal of Nutrition. 2015. [↑](#endnote-ref-11)
