# Supplementary material for: A simplified, combined protocol versus standard treatment for acute malnutrition in children 6–59 months (ComPAS trial): A cluster-randomized controlled non-inferiority trial in Kenya and South Sudan
Source: PLoS Med. 2020 Jul 9;17(7):e1003192. doi: 10.1371/journal.pmed.1003192 (PMC7347103; doi:10.1371/journal.pmed.1003192)
Supplement: Attachment — Submitted filename: Response letter_PLOS Med_2 May.docx [file pmed.1003192.s016.docx]

**Response to editor and reviewer comments**

Requests from the editors:

**Title – please provide the country setting and add in children aged 6-59 months**

We have added these two points to the paper title:

“A simplified, combined protocol versus standard treatment for acute malnutrition in children 6-59 months (ComPAS trial): a cluster randomized controlled non-inferiority trial in Kenya and South Sudan”

**The decimal point in numbers looks to be incorrectly formatted. Please do not place centrally and unbold.**

We searched the document but were not able to find any centrally placed or bolded decimal points. If we have missed something, we would be happy to correct this during the editing phase.

**In the abstract please state what the secondary outcomes are.**

Thank you for this suggestion. We have now added this information to the Abstract, Methods and findings, paragraphs 1 and 2.

**Data – please provide a link now and note that an author cannot be a point of contact for data access requests.**

The ComPAS dataset and supporting materials are available via the London School of Hygiene and Tropical Medicine data repository, and we have included this statement in our submission:

“Underlying data, code and supporting documentation for this paper are available at <https://datacompass.lshtm.ac.uk/1151/>. The full dataset cannot be made open access due to the presence of participant identifiable content. However, a redacted version will be provided to interested parties, subject to the completion of a request form and signing of a Data Transfer Agreement.”

This link has been added to the ‘Data sharing’ section at the end of the paper. It was emailed to PLOS Medicine on October 23, 2019, as an update to our original submission on October 1, 2019. Our original submission included the data files as supplementary materials.

**At this stage, we ask that you include a short, non-technical Author Summary of your research to make findings accessible to a wide audience that includes both scientists and non-scientists. The Author Summary should immediately follow the Abstract in your revised manuscript. This text is subject to editorial change and should be distinct from the scientific abstract. Please see our author guidelines for more information:** [**https://journals.plos.org/plosmedicine/s/revising-your-manuscript#loc-author-summary**](https://journals.plos.org/plosmedicine/s/revising-your-manuscript#loc-author-summary)

We have now added an author summary following the Abstract. We would be happy to accept any editorial changes.

**CONSORT checklist – please use sections and paragraphs instead of page numbers as these can change during revisions and formatting etc.**

We have updated both CONSORT checklists included with this manuscript: the checklist for non-inferiority and equivalence trials and the checklist for cluster randomized trials. These can be found as supplementary materials 6 and 7 in this submission.

Comments from the reviewers:

**Reviewer #1: This is a well-organized paper on a much awaited trial.**

Thank you for your review and helpful comments. We hope that our revisions now meet your approval for publication.

**Recovery rate in both ITT and per-protocol. In the  ITT it barely meets the Sphere standard for the combined protocol. In the per protocol- it is substandard. While the authors note the reasons for defaulting, it is exceptionally low. it also make me question the acceptability of the programs overall.**

We agree that the recovery rate was low, and defaulting was high in this trial. This was however similar in both arms because of contextual issues that affected both arms of the trial equally. The overall acceptability of the programs was not affected. There are two key reasons why the high rate of defaulting does not affect the interpretation or validity of our results:

- First, as is standard for non-inferiority trials, we designed our study with per-protocol as the main analysis (Piaggio et al 2012, Schumi et al 2011). Defaulters are not included in the per-protocol analysis, and the sample size was met for each cluster across each arm, therefore the intended power of this study was achieved. (Please note- the per-protocol rate of recovery for the combined protocol meets Sphere Standards; it is the intention-to-treat rate of recovery in both arms that did not meet Sphere standards).
- Second, in the ITT analysis where defaulters are included in the denominator, defaulting is high equivalently across both arms of the study. The contextual factors that drove up defaulting, which are described in the limitations section of the Discussion, paragraph 4, affected both arms equally and thus do not affect the between-arm comparisons this RCT was designed to assess. These issues include a nurse’s strike and political unrest during national elections in Kenya, and access to clinics during the rainy season in South Sudan.

We further address these issues in more detail in response to reviewer #2, comment #1 and #3 below. We have also added more detail on the per-protocol and ITT analyses, and the overall acceptability of the program, in the following sections of the paper:

- Abstract, Methods and findings, paragraph 1
- Author Summary
- Methods section, Statistical analysis, paragraphs 1 & 3
- Results section, paragraph 1
- Discussion section, paragraph 4

It is interesting to note that another similar nutrition study published recently in PLOS Medicine (Kangas et al 2019) also reported low rates of recovery in a randomized controlled trial (52.7% in the reduced dose arm and 55.4% in the standard dose arm; p = 0.45).

**The two contexts in the RCT are extremely different and this variation dies not come out in the summary statistics. While the study was powered to only produce a combined analysis, South Sudan is really quite different that then slums of Nairobi and the supplemental table 4 demonstrates those programming variations- for example length of stay. In terms of the cost effectiveness, I see this as significant limitation. However, the authors address the limitations in the discussion.**

As recognized, we are fully open about the two contexts being different and discuss this as a key limitation in the Discussion, paragraph 4. We did originally advocate for a larger study whereby each site would be independently powered, but the financial resources were not available to do so, as research funding in the humanitarian sector is limited.

To further address concerns related to country-specific differences, we present baseline characteristics by country in Supplementary Table 2, as well as exploratory analyses of recovery and length of stay by country in Supplementary Table 4. Because the study size is not powered for subgroup analyses, we include this as supplementary information rather than in the main text. We do however highlight some of the key baseline differences between the Kenya and South Sudan contexts within the manuscript, in the Results section, paragraph 2.

In terms of interpretation of the main conclusion in this paper- non-inferiority of recovery in the per-protocol analysis- these differences by context do not affect this conclusion because the randomization was stratified by country and the analysis adjusted for country. We describe this in further detail in response to reviewer 2, comment #3 below.

**Reviewer #2: Bailey et al present the results of a cluster randomized non-inferiority trial of a simplified, combine protocol for SAM and MAM compared to standard treatment. This is an important trial and addresses a subject of much discussion in the nutritional programming community. The authors describe this trail, which took place in Kenya and South Sudan with children enrolled in a total of 24 clinics. The authors find that the combined treatment for SAM and MAM is non-inferior to standard care concerning recovery. The authors also provide cost-effectiveness information.**

Thank you for your review. We hope we have adequately addressed your questions below.

**There is no doubt that addressing this question adds to the literature. That being said, the article requires some aspects to be more clearly addressed by the authors.**

**1)      There is an extraordinarily high loss to follow up (defaulting). As the authors state, this occurs in both arms, but is alarming. As such, the results, it could be argued, a limited to statement such as "among children remaining in the program, the two protocols were non-inferior." The differences between the ITT and PP analyses are such that this warrants a much larger discussion by the authors. Although balanced between arms, this is far above what would be acceptable in a program and limits what can be drawn from this trial.**

Please see our response to Reviewer #1, Comment #1, where we address the high defaulting, the differences between the ITT and PP analyses and the overall acceptability of the study and show where in the paper these clarifications can be found.

Expanding on that explanation, we designed this trial with per-protocol analysis as the main analysis because it is more conservative, requires a higher overall sample size and is the recommended approach for primary analysis of non-inferiority trials. We have added this information to the Methods, Statistical analysis, paragraph 3. ITT analyses are problematic in non-inferiority trials, as they make it easier to show non-inferiority (Piaggio et al 2012, Schumi et al 2011). Given our intended objective of this trial was to assess the non-inferiority of recovery in a per-protocol analysis, the defaulting does not limit what can be drawn from the trial, as defaulters were never intended to be included in the denominator. We have added this to the Discussion, paragraph 4. We expand on the issue of how the sample size was constructed, including the assumptions of defaulting, in response to Reviewer #2, Comment #2 below.

**2)      The randomization warrants further clarity. From what I understand, the 24 health centers were selected, and then randomized to one of two groups. Then, the statistician was blinded until the database was locked? This is all fine, but then the randomization is both neither complex and the study is open label. Shouldn't the analyses have been done under blind? Is that what the authors mean?**

We used stratified random sampling (stratification by country) to ensure control/intervention clusters in each country were balanced. This study is open-label, as blinding of trial participants and clinic-based researchers was not possible. The only blinding occurred at the level of the PI, to ensure objective trial management. Both of these points are described in the Methods, Randomization and blinding section, paragraph 1.

We implemented two key strategies to ensure impartial trial management, analysis and reporting:

- First, as the reviewer notes and as is now more clearly described in the Randomization and blinding section, paragraph 1, the principal investigator was blinded to treatment allocation until the last enrolled child completed treatment and the database was locked. The dataset and analysis do file are available via the LSHTM data repository. Once the database was locked, the PI was unblinded.
- Second, we did a double independent analysis. Both the PI and the senior statistician analyzed the data separately, using their own analysis files, to confirm and cross-check all results.

We have made some edits to the Methods, Randomization and blinding section, paragraph 1.

3)      **Although not typical, and this is greatly debated, I'm not entirely convinced about the power of this study given that the underlying assumptions for the ITT were very different than expected. It would be useful for the authors to comment a bit more on the analyses, provide additional information on lost to follow-up by cluster, and discuss further issues concerning the analyses themselves. This is also the case for the rationale for a cluster randomized trial, which is certainly warranted here, but doesn't come out clearly in the manuscript. Further to this, I would be more inclined to present the paper a bit more formally (was this a DSMB or not? It's unclear why Table 1 results are commented on? More details is needed in terms of the analyses**

We understand the reviewer’s concern about ITT analysis. This is an important point to clarify both in this response letter and in the manuscript. As described previously in response to Reviewer #1, comment # 1 and Reviewer #2, comment #1, the power and consequent validity of our study is not linked to ITT analysis. The power is instead based on the per-protocol analysis as the main analysis for this trial, and the underlying assumptions were used to calculate the sample size for a per-protocol analysis. Our primary objective was to assess non-inferiority of recovery in the per-protocol analysis. The ITT results were reported in keeping with best practices for reporting of non-inferiority trials (Piaggio et al 2012, Schumi et al 2011), which encourage reporting of both per-protocol and ITT. Therefore, the high defaulting rate (loss to follow-up) was controlled for by design, and the conclusion of non-inferiority for the primary outcome (recovery in the per-protocol analysis) was not affected by the loss to follow-up. More detail has been added to the Methods, Statistical analysis, paragraph 3 and Discussion, paragraph 4 on these points.

To provide more detail on the underlying assumptions, the sample size was calculated to ensure we recruited enough children per cluster so that when defaulters (and other non-adherents, such as transfers or early discharges) were removed from the denominator the statistical power to investigate non-inferiority would not be affected. We required 100 children per cluster (after exclusion of defaulters and other non-adherents) to ensure we achieved 80% power, therefore we aimed to recruit a minimum of 150 children per cluster to account for an expected high loss to follow-up. More detail on the sample size has been added to Methods, Statistical analysis, paragraph 1. This sample size was achieved; after data cleaning, the combined protocol arm had a median sample per cluster of 107.5, (IQR 99.5-118); the standard protocol arm had a median sample per cluster of 102.4, (IQR 97-107.5). We have added detail in the Results, paragraph 1, on the numbers retained by cluster.

We have added the rationale for the cluster randomized trial (as well as the choice to test for non-inferiority) in the Methods, Study design section.

We were advised by the LSHTM committee who provided oversight to this trial not to conduct a formal DSMB. This was because the duration of recruitment was expected to be short. These assumptions are described in our study protocol (included in this submission as Supplementary material 2 (S2)), Section VI, Methods: Monitoring, 20-Data Monitoring, 20a-Role of Data Safety and Monitoring Board and published in our study protocol paper (Bailey et al 2018). On recommendation of the LSHTM ethics committee we were advised to set up an Independent Trial Safety Committee. The Trial Safety Committee reviewed the study outcomes and adverse events (hospitalizations and deaths) at the mid-point of recruitment and determined there was no evidence of harm related to the trial, so the trial was deemed safe to continue. We have added more information about why we did not set up a formal DSMB and who comprised the trial safety committee in Methods, Outcomes, paragraph 1. Finally, we also wish to note that a full description of the methods for this trial are described in a separate protocol paper (Bailey et al 2018); this paper is referenced throughout the manuscript.

We only briefly comment on the results in Table 1 because this is a baseline table giving an overview of key characteristics of the study participants. We do not go into more detail because these characteristics are similar across the study arms. We do not provide statistical tests of significance on the difference in baseline characteristics between study arms because any differences should be accounted for by the randomized design. As recommended by CONSORT and discussed in the literature on RCTs (Moher et al 2010, de Boer et al 2015), it is not standard practice to include p-values in a table of baseline characteristics. We describe this in more detail in response to Reviewer #3, comment #2 below. We do however adjust for the baseline characteristics of age, sex and country in the analyses of outcomes (Table 2). We have added more detail to our description of the analyses and techniques used in Methods, Statistical analysis, paragraph 3.

**Minor comments**

*       **Some of the references are duplicated**

Apologies for this. We realized that reference #27 (Goossens et al 2012) was duplicated after submission and have now replaced it with an additional study that builds on the same work (Burza et al 2015). We also realized during the revision of this paper that references #17 and #50 were the same, so we have removed reference #50. Finally, we replaced Ashworth 1969 with Ashworth 2006, as the conclusions in the later paper incorporate those found previously and include additional evidence.

*       **The introduction could be updated to provide more recent and comprehensive information**

We have updated the Introduction, paragraph 4 to reflect the very recent publication of Daures et al 2020, an important operational study which evaluated the impact of an integrated SAM/MAM protocol in Burkina Faso. The Daures study used a dosage protocol based on a combination of MUAC and weight, and found an overall recovery rate of 86.3% (95% CI 85.4, 87.2) for children admitted with a MUAC<125mm and/or oedema, treated with a gradually reduced dosage or RUTF. Children with a MUAC<115mm or oedema received 175 kcal/kg/day of RUTF but recovery was lower in this group (70.4% (95% CI 67.5, 73.5).

In light of the current coronavirus pandemic, we have also added a description of how this work ties into the global health response to COVID-19 in the Discussion section, final paragraph. The onset of the pandemic has necessitated a consideration of simplified, easy-to-use and cost-effective nutrition treatment approaches. UNICEF is for example recommending the protocol studied in this trial as part of its adapted approach for treating malnutrition in communities affected by COVID-19 (<https://www.nutritioncluster.net/sites/default/files/2020-04/Wasting%20Programming%20COVID19%20Brief%201%20%28Draft%29_27%20March_v1.pdf>). We hope the publication of this paper will support current responses, as well as provide a well-cited baseline for future work in this area.

*       **Some of the definitions are unclear, median? Z-score?**

We have added more detail to the anthropometric definitions and reference to the WHO child growth standards in the Introduction in paragraph 2 and Statistical analysis section, paragraph 3, and have added a new section to the Methods, Study procedures section, paragraph 1 that refers to Supplementary table 1, where the admission criteria for each study arm are detailed.

***       The cost-effectiveness analysis is interesting, but it seems a bit less attention is paid to it in terms of framing this in a context of so many lost-to-follow up.**

We used the ITT sample to assess cost-effectiveness in order to give the full picture of cost in this trial, and the high loss to follow-up drives down potential cost savings. As cost-effectiveness is a secondary outcome, we do not assess non-inferiority for this outcome. We have now added more context for the interpretation of the cost results in the Discussion section, paragraphs 6 and 8, and have re-organized the discussion around cost to make it more coherent and to connect it more clearly to the issues of loss-to-follow-up (defaulting), country-specific cost differences, and program vs. research costs in humanitarian settings.

**Reviewer #3: The authors have reported the results of a recent, large non-inferiority cluster-RCT comparing the effectiveness of a combined protocol against standard care of ready to use therapeutic foods to treat sever and moderate acute malnutrition for 6-59 month old children in Kenya an South Sudan. They have found that the simplified combined protocol was non-inferior to standard protocol as defined by their primary outcomes of difference in proportion who experienced nutritional recovery. The results are convincing and important, which will likely have impact to change practice, given the large sample size, recruitment across both rural and urban settings, and the base case economic analyses show that the simplified protocol is also cheaper.**

**The manuscript is well-written, methodological sound in terms of recruitment methods, randomisation and analysis. I have very little critique and commend the authors on an impressive trial in a challenging environment. The recency of the trial also highlights a very important topic area of global significant with a large number of children suffering from acute malnutrition, in particular areas of Africa, leading to a high rate of morbidity of mortality. A simplified approach to nutrition supplementation may make it easier for clinics to operationalise and implement this approach in diverse settings. Future research should explore implementation research of the approach.**

Thank you for your review and kind support of our research. Indeed, we also hope that our paper helps inspire future work, both in program settings and also more formal implementation research as suggested.

**Overall I have very little critique of the paper. My only minor comments are:

Methods - description of the analysis: Generally, cluster RCT analyses are a bit more complex - outcomes at follow-up can be analysed either at aggregrate level cluster level of individual outcomes, with both cases adjusting for baseline covariates. If individual level outcomes are analysed at follow-up (which it seems like this study) - then the analysis should allow for clustering using a mixed regression or generalised estimation equations. see Donner A, Klar N. Design and analysis of cluster randomization trials in health research. Arnold, 2000. It would be helpful if the authors could describe in method the type of model and regression technique they utilised. Line 226 simply states "regression analyses" which is too generic here.**

We used binomial regression models for binary outcomes and linear regression models for continuous outcomes. In both approaches we used clustered robust standard errors to adjust for repeated measures (clustering) within health facility. We adjusted for the baseline characteristics of age, sex and country in all the adjusted analyses. We have now added more detail to our description of these analyses in Methods, Statistical analysis, paragraph 3.

**Methods - covariate adjustment - I see that the analyses have been adjusted for clustering. It's not clear from the methods section whether baseline adjustments to differences in children anthropometry were considered and adjusted. Given the unit of randomisation of a Cluster-RCT in a group, analyses will often adjust for baseline given repeated nature of the outcomes and individual differences within groups, but if the authors did not they should rationalise why not (i.e. expected differences between groups were expected to be minimal at baseline).**

We did not anticipate any anthropometric differences between the clusters, because the health facilities within each stratification (i.e., country) were geographically close together and serving the same population, and screening/referral procedures were the same across all clusters. Exploring and adjusting for baseline differences in a randomized controlled trial is discouraged in the CONSORT reporting standards for RCTs (Moher et al 2010). Such adjustment could only be justified if baseline characteristics were prognostic of the outcome and at the same time related to treatment group. However, because of randomization, there are unlikely to be any baseline differences related to treatment group. In this trial there was a balance of baseline characteristics between arms as shown in Table 1.

As noted previously, we did adjust for country, age and sex in the analyses of the primary and secondary outcomes.

**Methods - it would helpful to specify that the z-scores were based on the WHO reference standards for growth charts.**

We have added this in the text in the Introduction, paragraph 2, in the Methods, Statistical analysis, paragraph 3.

**Results - subgroup analyses: Given there are two geographical groups: rural and urban - was there any thought given to a potential sub-group analyses in urban and rural localities (i.e. most mothers in Aweil East report no formal education where as mothers in Nairobi reported secondary level attainment).**

The rural vs. urban divide is accounted for in the exploratory analyses by country (Supplementary tables 2 and 4); this is because all of the children in the Kenya sub-group are in an urban context and all of the children in the South Sudan sub-group are in a rural context. We have added this distinction between the rural and urban settings and how they align with country in the Results, paragraph 2.

**Reviewer #4: Compas trial, Bailey et al.**

**This is an important trial and ditto manuscript and the authors should be congratulated on the large piece work and efforts put in this. I was impressed by the numbers in both groups, the non-inferiority as well as the costs saved.**

Thank you, we greatly appreciate this positive feedback – and the opportunity to respond to your comments to further improve our paper to benefit future readers/researchers/practitioners.

**Introduction: one of the major things that are missing in the current rationale and introduction is in lines 84-87, page 4: "Evidence indicates…recover (Refs) till "but a study….dosage". Since the other arguments to come up with a combined approach for MAM (without danger signs) and uncomplicated SAM make sense, the authors need to elaborate in the introduction what this "evidence" indicates. The 3 references mentioned (24, 25, 26) should be briefly discussed or put into context. Did the authors in the papers/studies that are referred to use (in-)direct calorimetry, metabolic demands (RQ) to estimate and measure energy expenditure and usage? Was this done in the pre- or post RUTF era and were the study participants MAM or SAM patients? Lastly, it is not clear at present 'why' one would want to have a reduced dosage (line 87). Since all these arguments are underlying the study rationale, this needs more explanation to better put this trial into perspective.**

We have now added more detail to the descriptions of rate of growth and energy requirements referenced in the Introduction, paragraph 4, in order to make the background evidence and rationale clearer. However, to keep the background section of the Introduction concise and focused on the main messages, we summarize the key points and refer to the studies where readers can find more detail. These studies are described in more detail below.

Four studies assessed children in outpatient care with the use of RUTF for SAM. Goossens et al 2012, Binns et al 2015 and Burza et al 2015 evaluated the treatment of SAM in an outpatient setting with RUTF and found that MUAC and weight gain track each other during treatment. In 2015-2016, we conducted our own secondary analysis of children recovering from both SAM and MAM in five countries, and our findings were consistent with the Goossens, Burza and Binns studies. Rate of MUAC and weight gain mirrored each other and slowed as children recovered. Preliminary data from our ‘stage 1’ work was published in the technical magazine ‘Field Exchange (Bailey et al 2016),’ prior to commencing the field trial described in this paper. The full paper has been provisionally accepted for publication in PLOS One and we hope to include the full citation in the finalized paper. In our stage 1 study, energy requirements were indirectly estimated using a formula derived from the FAO 2001 Human Energy Requirements report, and we found that 1000 kcal/day meets the needs of children with a MUAC <12.5cm.

The Ashworth 2006 study reviewed the effectiveness of SAM treatment in different community settings and reported that rates of weight gain decrease as children recover and are lower in outpatient settings compared with inpatient settings.

The rationale for a reduced dosage is to increase coverage of treatment in a resource-constrained environment, given that the current dose (up to 200 kcal/kg/day in most SAM treatment programs) is assumed to be an over-estimation of energy needs. Energy requirements for catch up growth assume that maintenance requirements are 80-100 kcal/kg and the cost of growth is about 5 kcal /g. The estimation of 200 kcal/kg/day was developed when treatment was confined to inpatient settings and corresponds to a catch-up growth of around 20 g/kg/day (100 + 5 x 20), a rate of weight gain never achieved in outpatient settings (which are typically closer to 5g/kg/day). Several studies have now tested reduced dosage protocols (Maust et al 2015, James et al 2015, Kangas et al 2019, Daures et al 2020) as it is assumed children do not eat 200 kcal/kg/day in an outpatient setting. To make the best of available resources – which are sadly insufficient in most settings where SAM and MAM are common - programs need to find the right balance between individual energy needs (and ensuring the vast majority of children receive more than enough energy to recover) and a public health approach that meets the needs of more children. In our stage 1 study (Bailey et al 2016), we looked at this from an individual biological point of view (estimating energy needs for children recovering from acute malnutrition and how this correlates with where children are anthropometrically), and a public health point of view (what is the optimal dosage that meets needs but stretches resources further).

We have added more detail to the rationale for a reduced dosage in the Introduction, paragraph 4.

**Page 5, Participants: Line 120: it would be good to know the % of children that were enrolled via active case finding and the % that were enrolled without. Do the authors feel this might have been a source of bias?**

We did not collect specific information about the proportion of children enrolled via active case finding vs. the proportion enrolled without it. While we agree children found by active case finding may be different than those who present to health facilities on their own, we do not believe that this would have affected results or overall interpretation because:

- Active case finding was conducted similarly in all clinics and in both countries.
- Randomization ensured any between-clinic differences were distributed equally across the arms.

**P6, line 139: please clarify of the PI did all data-analyses and data interpretation?**

As noted in response to Reviewer #2, comment #2, we did a double independent analysis. Both the PI (JB) and the senior statistician (CO) analyzed the data separately, using their own analysis files, to confirm and cross-check all results. Another co-author (NL) analyzed the cost effectiveness data presented in tables 3 & 4, which were cross-checked and confirmed by the PI and senior statistician. The ‘contributor’ section at the end of the manuscript notes that JB, CO and NL analyzed the data.

**Same page, line 145: the section on study procedures is quite difficult to read and comprehend and it took me 3 times to understand the work-flow.**

We have edited the section on Study procedures in this paper to improve readability. We hope that this is now clearer and easier – we are happy for editors to suggest further changes in a final proof version.

As noted previously, we published a protocol paper in BMC Trials in 2018 which gives more detail on the study procedures (Bailey et al 2018).

**Based on what evidence are the recommendations regarding intake done ("Combined protocol clinics….to 1 sachet RUTF/day (line 149)".**

The dosage protocol tested in this trial was developed during the ‘ComPAS stage 1’ work, as described in preliminary form in Field Exchange magazine (Bailey et al 2016) and currently pending publication in PLOS One. As noted at the end of paragraph 4 in the Introduction, ‘We previously conducted a secondary analysis of program data from five countries and found that children recovering from acute malnutrition with a MUAC<12.5cm may require 1,000 kcal per day; a reduced dosage compared to standard care.’ The reduction in dosage once children reach 11.5cm (from 2 sachets RUTF/day to 1 sachet RUTF/day) is based on the plateau in growth rate and corresponding decrease in supplemental energy requirements that we observed at approximately 11.5cm. This also aligns with UN guidance on the treatment of MAM (WHO Technical note: supplementary foods for the management of moderate acute malnutrition in infants and children 6–59 months of age 2012, MAM Decision Tool for Emergencies 2017), which recommends 1 sachet RUSF/day (500-550 kcal/day) for children with a MUAC of 11.5-<12.5cm. We have added more information on this rationale in the Introduction, paragraph 4 and in the Methods, Study procedures, paragraph 1.

The Introduction, paragraph 4 summarizes some of the key studies that have tested a reduction in dosage as children progress through treatment, which provide additional rationale for the reduction from 2 sachets to 1 sachet/day once children reach a MUAC of 11.5cm (Maust et al 2015, James et al 2015, Kangas et al 2019, Daures et al 2020).

**Page 14: since this is underlying the main result, that there is no difference between the 2 protocols and hence non-inferiority; I would recommend stressing that there was no difference between the ITT analysis versus the PP analysis.**

We agree the outcomes of both analyses reinforce each other. As suggested, we have now emphasized in the Discussion, paragraph 1, that ITT analysis is consistent with conclusions arising from the PP analysis.

**Page 19, last few sentences of the results sections; the 'exploratory analyses' show that subgroups recovered similarly. This is (in addition to my previous comment) an important finding and can (should) be presented a bit more prominently than is currently done.**

We have added more to highlight this point in the Results, paragraph 9 and the Discussion, paragraph 1. Of note, no matter how we break-up the data in various sub-groups, we still see the same overall patterns. The reviewer may be interested to know that we are currently working on a secondary analysis paper of concurrently wasted-stunted children in the ComPAS trial, to be submitted once this paper is published, and similar recovery between the study arms can be seen within these sub-groups as well.

**The discussion reads well and is to-the-point. It would be interesting to elaborate a bit more on your ideas (lines 391-394) on the 'future research' you suggest. Although I think you are right here it would be good from an academic perspective to hear your thoughts were in the spectrum from MAM to complicated SAM the combined protocol might still work and were it might stop being effective (non-inferior) to have 1 combined protocol.**

Thank you. This is an important point to reflect on, and we agree that readers would be interested in some more detail. We have added more detail to this in the Discussion, paragraph 9. We believe that in contexts where children are highly vulnerable to malnutrition- particularly in crises and chronically fragile situations- the combined protocol may offer some protection to children enabling them to avail of treatment before they reach the more severe stages of malnutrition. The optimal diagnostic criteria and dosage of therapeutic foods may change by setting. We hope our research inspires others to investigate where and when simplified, combined protocols such as this can be most of use.

**References**

Ashworth A. Growth rates in children recovering from protein-calorie malnutrition. Brit J Nutr 1969; 23:835

Bailey J, Chase R, Kerac M, Briend A, Manary M, Opondo C, et al. Combined protocol for SAM/MAM treatment: the ComPAS study. Field Exchange 2016; 53(44–46).

Bailey J, Lelijveld N, Marron B, Onyo P, Ho LS, Manary M, et al. Combined Protocol for Acute Malnutrition Study (ComPAS) in rural South Sudan and urban Kenya: study protocol for a randomized controlled trial. Trials 2018; 19(1): 251.

Binns P, Dale N, Hoq M, Banda C, Myatt M. Relationship between mid upper arm circumference and weight changes in children aged 6-59 months. Arch Public Health 2015; 73:54.

Daures, M., et al., New approach to simplifying and optimising acute malnutrition treatment in children aged 6-59 months: the OptiMA single-arm proof-of-concept trial in Burkina Faso. Br J Nutr, 2020. **123**(7): p.756-767.

de Boer MR, Waterlander WE, Kuijper LD, Steenhuis IH, Twisk JW. Testing for baseline differences in randomized controlled trials: an unhealthy research behavior that is hard to eradicate. Int J Behav Nutr Phys Act. 2015 Jan 24;12:4. doi: 10.1186/s12966-015-0162-z. PMID: 25616598; PMCID: PMC4310023.

Global Nutrition Cluster: MAM Task Force. MAM Decision Tool for Emergencies. 2017. Geneva: IASC Global Nutrition Cluster.

Goossens S, Bekele Y, Yun O, Harczi G, Ouannes M, Shepherd S. Mid-upper arm circumference based nutrition programming: evidence for a new approach in regions with high burden of acute malnutrition. PLoS One 2012. 7(11): e49320.

Burza

James PT, Van den Briel N, Rozet A. Low‐dose RUTF protocol and improved service delivery lead to good programme outcomes in the treatment of uncomplicated SAM: a programme report from Myanmar. Maternal & Child Nutrition 2015; 11:859-869.

Kangas ST, Salpéteur C, Nikièma V, Talley L, Ritz C, Friis H, et al. Impact of reduced dose of ready-to-use therapeutic foods in children with uncomplicated severe acute malnutrition: A randomised non-inferiority trial in Burkina Faso. PLOS Medicine 2019; 16(8): e1002887. <https://doi.org/10.1371/journal.pmed.1002887>.

Maust A, Koroma A, Abla C, Molokwu N, Ryan KN, Singh L, et al. Severe and moderate acute malnutrition can be successfully managed with an integrated protocol in Sierra Leone. J Nutr 2015; 145(11): 2604-9.

Moher D, Hopewell S, Schulz K, Montori V, Gotzsche P, Devereaux P J, Elbourne D, Egger M, Altman D. Consort 2010 Explanation and Elaboration: updated guidelines for reporting parallel group randomized trials. BMJ 2010; 340:c869.

Piaggio G, Elbourne DR, Pocock SJ, Evans SJ, Altman DG, Consort Group. Reporting of noninferiority and equivalence randomized trials - extension of the CONSORT 2010 statement. JAMA 2012; 308(24):2594-2604.

Schumi J and Wittes JT. Through the looking glass: understanding non-inferiority. Trials 2011; 12(1):106.

The World Health Organization. Technical note: supplementary foods for the management of moderate acute malnutrition in infants and children 6–59 months of age. Geneva, World Health Organization, 2012.
